# Loss of Proteostasis and Early-Onset Neurodegeneration in Down Syndrome: From Mechanisms to Interventions

**DOI:** 10.3390/antiox15040520

**Published:** 2026-04-21

**Authors:** Antonella Tramutola, Chiara Lanzillotta, Fabio Di Domenico, Eugenio Barone, Marzia Perluigi

**Affiliations:** Department of Biochemical Sciences “A. Rossi-Fanelli”, Sapienza University of Rome, Piazzale A. Moro 5, 00185 Roma, Italy; antonella.tramutola@uniroma1.it (A.T.); chiara.lanzillotta@uniroma1.it (C.L.); fabio.didomenico@uniroma1.it (F.D.D.); eugenio.barone@uniroma1.it (E.B.)

**Keywords:** Down syndrome, proteostasis, oxidative stress, mitochondrial dysfunction, Alzheimer’s disease

## Abstract

Down syndrome (DS), caused by trisomy 21, is the most prevalent genetic condition associated with accelerated aging and near-universal development of early-onset Alzheimer’s disease (AD). Beyond gene-dosage imbalance, trisomy 21 induces widespread transcriptional, metabolic, and proteomic remodeling that establishes a chronic state of proteotoxic and oxidative stress from early development. Increasing evidence identifies DS as a disorder of proteostasis network failure, in which sustained translational pressure, redox disequilibrium, and degradation pathway insufficiency progressively erode cellular resilience. In the DS brain, persistent endoplasmic reticulum stress with PERK-dominant signaling, mitochondrial dysfunction characterized by oxidative phosphorylation deficits and excessive reactive oxygen species production, and impaired antioxidant responses create a highly vulnerable intracellular environment. Concomitantly, degradation systems become compromised: proteasomal catalytic activity declines, ubiquitin-dependent signaling is remodeled, and chronic mTOR hyperactivation suppresses autophagic and mitophagic flux. The coordinated impairment of the ubiquitin–proteasome system and autophagy establish a feed-forward cycle of proteotoxic accumulation and redox amplification. Within this framework, Alzheimer-like neuropathology in DS emerges not solely from amyloid precursor protein triplication but as the late manifestation of decades-long proteostasis exhaustion. Therapeutic strategies aimed at restoring global proteostasis and redox balance may therefore represent a more effective systems-level approach to mitigating neurodegeneration in DS.

## 1. Introduction

Down syndrome (DS), caused by full or partial trisomy of chromosome 21 (Chr21), is the most common chromosomal aneuploidy compatible with postnatal survival and the leading genetic cause of intellectual disability [1]. Its prevalence varies slightly by region, but it occurs in approximately 1 in every 700–1000 live births globally. Advances in prenatal screening and maternal age trends have influenced these rates, as the likelihood of having a child with DS increases with maternal age. Despite variations, it remains a relatively common genetic condition, with millions of individuals affected worldwide, and improved healthcare has significantly increased life expectancy and quality of life for DS individuals. As survival has improved, it has become increasingly evident that DS is not merely a neurodevelopmental disorder, but a systemic and progressive condition characterized by premature aging and early emergence of age-associated comorbidities. Clinically, DS presents with intellectual disability and a broad constellation of multisystem alterations—including congenital heart defects, thyroid dysfunction, immune dysregulation, gastrointestinal anomalies, metabolic disturbances, and increased susceptibility to hematological malignancies. However, this phenotypic heterogeneity reflects a deeper biological principle: trisomy 21 does not simply increase the expression of individual genes but reshapes regulatory networks across the genome [2]. The additional chromosome imposes genome-wide transcriptional, epigenetic, and proteomic remodeling that alters developmental trajectories and systemic homeostasis from embryogenesis onward [3]. At the molecular level, overexpression of more than 200 Chr21 genes perturbs chromatin organization, modifies DNA methylation patterns, alters histone marks, and reshapes non-coding RNA networks [4]. These changes extend beyond proportional transcript amplification, affecting global gene expression programs involved in metabolism, immune signaling, mitochondrial function, and cellular stress responses. Consequently, trisomic cells operate in a state of persistent regulatory disequilibrium. Increased gene dosage elevates global protein synthesis and disrupts stoichiometric relationships within multiprotein complexes, creating a chronic imbalance between protein production, folding, and degradation. This sustained pressure on protein quality control systems establishes a proteotoxic background that intersects with redox imbalance and metabolic dysregulation [5]. The central nervous system (CNS) is particularly vulnerable to these disturbances. Neurodevelopment in DS is characterized by reduced neural progenitor proliferation, altered neuronal differentiation, impaired synaptogenesis, and diminished dendritic arborization, leading to reduced brain volume—especially within the hippocampus and frontal cortex. These structural abnormalities unfold within a cellular environment marked by mitochondrial dysfunction, increased oxidative stress, and chronic low-grade inflammation. Triplication of interferon receptor genes enhances innate immune responsiveness, while overexpression of redox-regulating genes such as SOD1 perturbs antioxidant balance [6]. The convergence of these mechanisms establishes a stress-prone neural milieu that predisposes to premature biological aging. Among the most consequential gene-dosage effects is triplication of the amyloid precursor protein (APP) gene, which confers an almost inevitable risk of early Alzheimer-related neuropathology [7]. Yet Alzheimer’s disease (AD) in DS cannot be attributed solely to amyloid overproduction. Amyloid-β accumulation unfolds within a broader landscape of progressive mitochondrial fragility, impaired proteostasis, chronic oxidative stress, and diminished adaptive stress responses. Thus, AD pathology in DS emerges not as an isolated consequence of APP dosage [8], but as the downstream manifestation of long-standing cellular disequilibrium.

Individuals with DS represent the most prevalent genetically determined form of early-onset Alzheimer’s disease (EOAD), with nearly universal development of AD neuropathology by the fourth decade of life [9]. However, amyloid accumulation in DS occurs within a biological context of systemic and brain-specific accelerated aging. Trisomy 21 establishes a persistently stress-prone cellular state decades before chronological aging would predict, lowering resilience thresholds and amplifying the neurotoxic effects of amyloid-β (Aβ) and tau pathology. Beyond APP triplication, overexpression of Chr21 genes such as SOD1, DYRK1A, and RCAN1 contributes to dysregulation of redox signaling, kinase activity, mitochondrial function, and calcium homeostasis [10]. These alterations promote sustained oxidative stress, metabolic instability, and impaired stress adaptation. Mitochondrial dysfunction emerges as a central vulnerability node, characterized by impaired oxidative phosphorylation, altered mitochondrial dynamics, defective mitophagy, and excessive production of reactive oxygen species (ROS) [11]. Bioenergetic insufficiency not only compromises neuronal function but also weakens energy-dependent proteostasis mechanisms, reinforcing a feed-forward cycle between redox imbalance and proteotoxic stress. Persistent oxidative and metabolic stress promotes premature activation of cellular senescence programs across neurons and glial populations. Senescent cells exhibit activation of p53/p21 and p16^INK4a^ pathways, chronic DNA damage signaling, mitochondrial impairment, and extensive epigenetic remodeling [12]. A hallmark of senescence is progressive loss of proteostasis capacity, including impaired autophagic flux, reduced proteasomal activity, and maladaptive unfolded protein responses [13]. In this context, mitochondrial dysfunction limits ATP availability for protein folding and degradation, while oxidative modifications increase the aggregation propensity of Aβ and tau. The senescence-associated secretory phenotype (SASP) further sustains inflammatory signaling and activates tau kinases, directly linking premature cellular aging to synaptic dysfunction and network instability [14]. Neuropathologically, AD in DS mirrors sporadic AD in its anatomical progression but develops decades earlier. Amyloid deposition begins in early adulthood, followed by widespread tau pathology by the fourth decade, whereas clinical dementia manifests later as compensatory mechanisms progressively fail [15]. Biomarker studies—including cerebrospinal fluid analyses and PET imaging—demonstrate a prolonged preclinical phase during which mitochondrial dysfunction, oxidative stress, and proteostatic insufficiency silently accumulate. This predictable and genetically defined trajectory positions DS as a powerful human model for dissecting the interplay between accelerated aging and neurodegeneration [16].

Despite the near-universal development of AD neuropathology in individuals with DS, there is substantial inter-individual variability in the timing and clinical manifestation of dementia. This heterogeneity suggests that genetic dosage alone is insufficient to fully explain disease expression and highlights the contribution of modifying factors that confer resistance or resilience. Emerging evidence indicates that socio-behavioral, lifestyle, and environmental determinants play a critical role in shaping cognitive trajectories in DS. Factors such as cognitive stimulation, social engagement, physical activity, sleep quality, and access to structured care environments have been associated with delayed onset or reduced severity of dementia symptoms, even in the presence of significant neuropathological burden [17]. These domains interact dynamically across the lifespan and are closely intertwined with biological processes central to DS pathophysiology, including proteostasis, redox balance, and metabolic regulation. For example, physical activity and environmental enrichment can modulate mitochondrial function, enhance proteostatic capacity, and attenuate neuroinflammatory signaling, while social and cognitive engagement may contribute to the maintenance of neural network integrity and cognitive reserve. Conversely, adverse environmental exposures and chronic stress can exacerbate oxidative and proteotoxic stress, lowering resilience thresholds. Within this framework, resilience to AD in DS emerges as a systems-level property resulting from the cumulative interaction between genetic vulnerability and modifiable life-course factors, rather than from a single protective mechanism. This perspective aligns with recent models emphasizing the dissociation between neuropathology and clinical expression and underscores the importance of integrating biological and socio-environmental dimensions to better understand disease variability and to inform preventive and therapeutic strategies. Variability in resilience can be interpreted as the result of differential capacity to maintain proteostasis and redox balance under chronic trisomy-driven stress, thereby linking life-course modifiers to the molecular mechanisms of proteotoxic vulnerability described below.

## 2. Trisomy 21 and Proteotoxic Stress

Proteostasis is maintained by a highly integrated and dynamically regulated network of pathways that cooperate to preserve protein quality and functionality. Molecular chaperones assist in the folding and refolding of nascent or misfolded proteins, but when refolding is unsuccessful, these substrates are selectively directed toward degradation systems such as the ubiquitin–proteasome system (UPS) or the autophagy–lysosome pathway. Importantly, these components are not independent; rather, they are extensively cross-regulated at multiple levels [13]. For instance, chaperones not only facilitate folding but also act as triage factors, determining whether a protein should be refolded or targeted for degradation. Similarly, impairment or saturation of the UPS can trigger a compensatory upregulation of autophagy, ensuring the clearance of protein aggregates that exceed proteasomal capacity [18].

This coordination is further controlled by stress-responsive signaling pathways, including the unfolded protein response (UPR) and the heat shock response (HSR), which transcriptionally and post-translationally modulate all arms of the proteostasis network. These pathways can simultaneously enhance chaperone expression, suppress global protein synthesis, and increase degradation capacity, thereby redistributing cellular resources under stress conditions. Moreover, feedback loops exist whereby the accumulation of misfolded proteins directly influences the activity of these signaling pathways, reinforcing adaptive responses. Through this multilayered cross-talk, the proteostasis network operates as a flexible and adaptive system [19].

However, when the balance between folding capacity, degradation efficiency, and stress signaling is disrupted, this coordination fails, ultimately predisposing the cell to proteotoxic stress [20].

As introduced above, trisomy 21 imposes a chronic imbalance between protein synthesis, folding, and degradation, establishing a proteotoxic background [21].To understand how this imbalance arises, it is necessary to examine the molecular architecture of the proteostasis network. Increased gene dosage elevates global protein synthesis and disrupts the stoichiometric balance required for correct assembly of multiprotein complexes. Proteins encoded on chromosome 21 frequently require binding partners located on other chromosomes; when produced in excess, they remain unassembled or misfolded, increasing the burden on molecular chaperones and degradation pathways [22]. This imbalance establishes a chronic proteostatic strain in which stress-response pathways remain persistently engaged. Proteotoxic stress in DS is further amplified by mitochondrial dysfunction and redox imbalance. Oxidative modifications destabilize protein structure and promote aggregation, while impaired ATP production compromises the efficiency of energy-dependent clearance systems, including the ubiquitin–proteasome system and autophagy–lysosome pathways. Rather than reflecting isolated pathway failures, proteostasis disruption in DS represents a network-level disturbance in which increased protein synthesis, defective degradation, oxidative stress, altered translational control, and organelle dysfunction converge [23]. Neurons are particularly susceptible to this chronic imbalance due to their high metabolic demand, complex proteome dynamics, and limited regenerative capacity. Over time, sustained proteostatic insufficiency reduces synaptic protein turnover, weakens adaptive stress responses, and lowers the threshold for aggregation-driven toxicity [24]. Aging superimposed on this pre-stressed background further diminishes buffering capacity, accelerating the transition from compensatory stress signaling to irreversible neurodegeneration. Viewed through this integrative lens, DS emerges as a paradigmatic condition in which gene-dosage imbalance drives chronic proteotoxic stress, accelerates biological aging, and creates a permissive environment for Alzheimer-like pathology. The following sections will dissect how endoplasmic reticulum stress, mitochondrial dysfunction, and degradation pathway impairment interact within a unified proteostasis–redox network and will explore therapeutic strategies aimed at restoring systemic resilience rather than targeting single pathogenic proteins.

### Endoplasmic Reticulum Stress and Unfolded Protein Response in Down Syndrome Neuropathology

Trisomy 21 imposes a chronic proteotoxic burden that persistently engages endoplasmic reticulum (ER) stress signaling and reshapes unfolded protein response (UPR) dynamics in DS. Rather than representing a transient adaptive reaction, ER stress in DS appears developmentally established and progressively maladaptive. The ER, a central hub for protein folding, calcium homeostasis, and post-translational modification, is highly sensitive to disturbances in redox balance and proteome load. In trisomic cells, increased protein synthesis, oxidative modifications, and altered calcium handling converge to destabilize ER proteostasis, promoting accumulation of misfolded proteins and sustained activation of UPR pathways [25,26]. Although the UPR is classically defined as a conserved adaptive program that reduces translation and enhances folding capacity [27], its chronic engagement can shift from protective to deleterious, ultimately promoting apoptosis and functional decline [28]. Multiple independent lines of evidence indicate that ER stress and maladaptive UPR activation are embedded features of DS neuropathology. Proteomic and biochemical analyses of human DS brain tissue demonstrate increased activation of ER stress sensors and dysfunction of key ER chaperones [29,30,31]. Accumulation of oxidatively modified proteins within the ER lumen activates the three canonical UPR branches—PERK, ATF6, and IRE1—via dissociation from the master regulator GRP78/BiP [26]. Notably, GRP78 itself is oxidatively modified in the DS brain [23], impairing its regulatory capacity and providing a direct mechanistic link between redox imbalance and sustained ER stress signaling [32]. Among UPR branches, the PERK pathway emerges as the dominant and persistently engaged arm in DS. Biochemical studies of frontal cortex from individuals with DS reveal chronic activation of the PERK/eIF2α/ATF4 axis [30]. PERK-mediated phosphorylation of eIF2α reduces global protein synthesis, thereby limiting ER client load [33], while selectively enhancing translation of stress-responsive transcripts such as ATF4 [34]. Under physiological conditions, ATF4 coordinates adaptive outputs, including induction of chaperones, redox-regulatory genes, autophagy components, and amino acid metabolism pathways. Negative feedback through GADD34 allows re-establishment of translational homeostasis [35]. In DS, however, this regulatory balance appears disrupted. Reduced GADD34 levels and persistent eIF2α phosphorylation indicate failure to restore translational equilibrium, leading to sustained activation of pro-apoptotic mediators such as CHOP and BCL-2 family members [36]. Importantly, PERK hyperactivation in DS is closely associated with Alzheimer-like neuropathological features. Both young and older DS brains exhibiting AD pathology show increased pPERK, peIF2α, ATF4, and CHOP levels [30], paralleling observations in sporadic AD, where ER stress markers correlate with Braak stage progression [30,37,38,39,40]. These findings suggest that PERK-dominant UPR signaling is not a secondary epiphenomenon but a central contributor to neurodegenerative vulnerability. A critical pathogenic feature in DS is the uncoupling between PERK activation and antioxidant defense. Under normal conditions, PERK signaling promotes nuclear translocation of Nrf2, enhancing antioxidant gene expression. In DS, despite sustained PERK activation, Nrf2-driven transcription is suppressed [30]. This maladaptive dissociation is linked to overexpression of Bach1, a chromosome 21-encoded transcriptional repressor of Nrf2 targets [41,42], resulting in continued translational repression without adequate redox compensation. Consequently, the DS brain exhibits a state of PERK dominance combined with antioxidant insufficiency, amplifying oxidative damage and reinforcing proteostatic imbalance [43].

Systems-level proteomic analyses further support the centrality of ER stress in DS. Large-scale studies of post-mortem frontal cortices from young and aged individuals with DS reveal early and persistent dysregulation of stress-response and proteostasis pathways that intensify with aging and AD conversion [44,45,46,47]. Importantly, these alterations are detectable before overt neurodegeneration, indicating that UPR dysregulation is embedded within the DS molecular phenotype rather than representing a late-stage response. Energy metabolism, synaptic transmission, and cellular stress-response networks consistently emerge as the most affected pathway clusters, reinforcing the concept that chronic ER stress contributes to progressive neuronal dysfunction [44]. Proteostasis abnormalities in DS are not restricted to the CNS. Proteomic profiling of peripheral blood mononuclear cells (PBMCs) from children with DS demonstrates widespread dysregulation of metabolic, trafficking, and stress-response pathways, including activation of ER stress and UPR components [30,48]. Increased oxidative protein damage and sustained UPR signatures are detectable early in life, supporting the concept of a developmentally established “pre-stressed” proteostatic state. Similarly, lymphoblastoid cell lines derived from children with DS display persistent UPR induction and early endosomal alterations, consistent with systemic proteotoxic burden [30,49]. Broader analyses of the proteostasis network in DS cells confirm coordinated changes in chaperone systems, UPR mediators, and proteasomal machinery, alongside heightened sensitivity to additional proteotoxic challenges [45,48,50]. Reduced buffering capacity and lower stress tolerance thresholds suggest that trisomic cells operate closer to proteostasis collapse.

Murine models of DS provide temporal and mechanistic resolution of ER stress dynamics [51]. The Ts65Dn model exhibits early activation of ER stress pathways in the brain, with selective engagement of the PERK branch detectable prior to overt neurodegeneration [52]. Increased phosphorylation of PERK and eIF2α at young ages indicates that ER stress signaling is an upstream event in DS neuropathology. Longitudinal profiling reveals that UPR activation is not static; early hyperactivation may transition into partial signaling exhaustion or maladaptive remodeling with aging, consistent with progressive loss of stress-response efficiency [53,54]. This temporal evolution aligns with the broader concept of premature molecular aging in the DS brain.

Mechanistic validation is provided by studies in the Ts2Cje model, where pharmacological inhibition of PERK signaling restores translational balance, enhances Nrf2 nuclear translocation, and corrects Nrf2/Bach1 imbalance [30]. These interventions demonstrate that PERK hyperactivation is not merely correlative but causally linked to oxidative and proteostatic dysfunction, highlighting its therapeutic tractability. The more recently developed Ts66Yah model, which improves construct validity by eliminating non-Hsa21 orthologous regions [55], confirms early and persistent dysregulation of stress-response and proteostasis pathways across the lifespan [56]. Although not restricted to ER markers, proteomic analyses consistently identify UPR components among the most perturbed molecular signatures, reinforcing the centrality of chronic stress-response engagement in DS neuropathology [44]. Age-dependent cognitive decline in Ts66Yah mice parallels progressive redox imbalance and proteostasis disruption, supporting the notion that ER stress contributes to long-term functional vulnerability.

Beyond the canonical UPR, the integrated stress response (ISR) is also activated in DS. Costa-Mattioli and colleagues demonstrated phosphorylation of eIF2α in Ts65Dn hippocampus, post-mortem DS brain tissue, and DS-derived induced pluripotent stem cells (iPSCs), mediated in part by PKR activation [31]. Importantly, normalization of eIF2α phosphorylation restored proteostatic balance and improved cognitive performance in DS models [31], underscoring the pathological relevance of sustained translational repression.

Collectively, convergent evidence from the human brain, peripheral cells, and multiple DS mouse models positions ER stress and UPR dysregulation as central nodes in DS pathobiology. Trisomy 21 establishes chronic proteotoxic and oxidative pressure that persistently activates ER stress sensors, biases UPR signaling toward PERK dominance, uncouples antioxidant defense, and progressively undermines synaptic and neuronal integrity. While acute UPR activation may initially buffer proteome imbalance, sustained engagement—particularly of the PERK–ATF4–CHOP axis—promotes translational repression, pro-apoptotic signaling, and heightened vulnerability to Alzheimer-like neurodegeneration [57]. These findings collectively identify ER stress modulation, restoration of Nrf2 activity, and reinforcement of proteostasis capacity as rational therapeutic strategies to counteract neurodegenerative progression in Down syndrome [29,30,44].

## 3. Mitochondrial Stress in Down Syndrome

**Figure 1:**
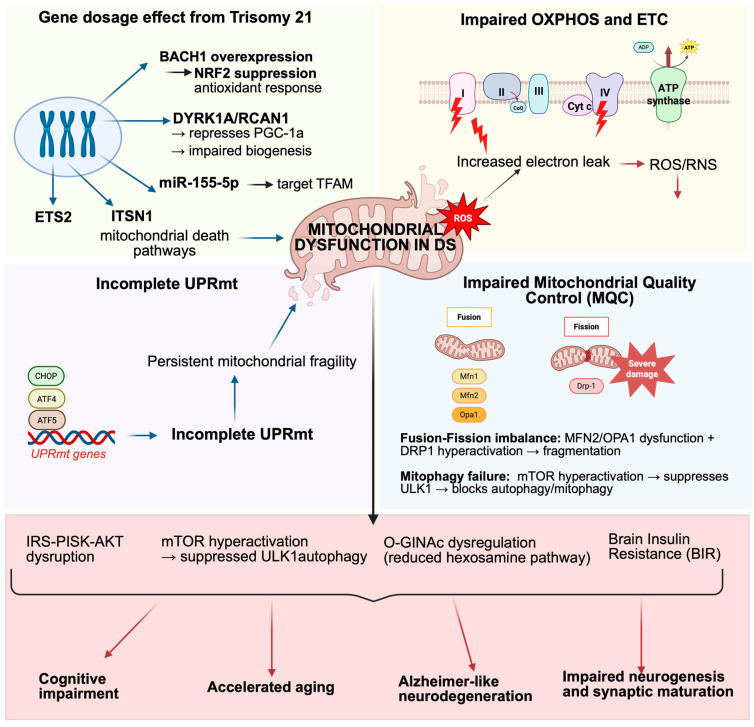
Mitochondrial dysfunction in Down syndrome. Schematic representation of the main mitochondrial alterations associated with trisomy 21. Gene dosage effects of chromosome 21-encoded products (ETS2, ITSN1, BACH1, DYRK1A/RCAN1 and miR-155-5p) impair mitochondrial biogenesis and antioxidant defenses, promote mitochondrial death pathways and contribute to persistent mitochondrial fragility. These primary defects lead to an incomplete mitochondrial unfolded protein response (UPRmt) and to impaired oxidative phosphorylation (OXPHOS), characterized by dysfunction of the electron transport chain (complexes I–IV and ATP synthase), increased electron leak and overproduction of reactive oxygen and nitrogen species (ROS/RNS). Altered mitochondrial quality control (MQC) further aggravates mitochondrial damage through fusion–fission imbalance (MFN2/OPA1 dysfunction with DRP1 hyperactivation and excessive fragmentation) and defective mitophagy driven by mTOR hyperactivation and suppression of ULK1-dependent autophagy. Together with IRS–PI3K–AKT pathway disruption, O-GlcNAc dysregulation (reduced hexosamine pathway) and brain insulin resistance, these alterations converge to drive cognitive impairment, accelerated aging, Alzheimer-like neurodegeneration, and impaired neurogenesis and synaptic maturation in individuals with Down syndrome.

In DS, mitochondria emerge as a central bioenergetic and redox hub through which trisomy-driven proteotoxic pressure is translated into progressive cellular dysfunction. Beyond their canonical role in ATP production, mitochondria regulate calcium buffering, redox signaling, lipid metabolism, and apoptotic pathways—functions that are particularly critical in neurons and glial cells with high metabolic demand and limited regenerative capacity [58]. In trisomy 21, mitochondrial destabilization leads to increased production of reactive oxygen and nitrogen species (ROS/RNS), propagating oxidative modifications of proteins and lipids, including carbonylation, nitration, and peroxidation-derived adducts [59,60]. Persistent oxidative stress damages mitochondrial DNA (mtDNA), respiratory chain components, and membrane integrity [61], further impairing electron transport chain (ETC) efficiency and reducing ATP output. This bioenergetic decline not only compromises neuronal function but also destabilizes energy-dependent proteostasis systems, amplifying protein misfolding and aggregation under oxidizing conditions [62]. Mitochondria therefore act as a mechanistic bridge linking gene-dosage imbalance to accelerated aging and Alzheimer-like neurodegeneration in DS [59,60,62,63,64]. Hallmarks of mitochondrial dysfunction in DS include reduced oxidative phosphorylation (OXPHOS) efficiency, chronic redox imbalance, and disruption of mitochondrial quality control (MQC) programs encompassing biogenesis, dynamics, and mitophagy [32,65] (Figure 1). These alterations are not restricted to the central nervous system; they contribute to systemic phenotypes such as cognitive impairment, congenital cardiac defects, and premature aging traits [1,60,65]. Importantly, mitochondrial dysfunction is detectable early in development, with widespread abnormalities reported in fetal trisomy 21-derived cells [66,67,68]. Structural fragmentation of the mitochondrial network, cristae disorganization, and reduced connectivity indicate chronic imbalance in mitochondrial turnover and distribution [69]. During neurogenesis and synaptic maturation—periods of intense bioenergetic demand—such instability is particularly detrimental, correlating with impaired neuronal proliferation, altered differentiation, reduced dendritic complexity, and increased susceptibility to apoptosis in DS models [64,70,71,72].

### 3.1. Mitochondrial Unfolded Protein Response (UPRmt) in Down Syndrome

The mitochondrial unfolded protein response (UPRmt) constitutes a critical adaptive program that attempts to buffer mitochondrial proteotoxic stress by coordinating retrograde signaling to the nucleus [73]. Upon accumulation of misfolded proteins within the mitochondrial matrix, UPRmt induces transcriptional programs that enhance expression of mitochondrial chaperones, proteases, antioxidant defenses, and metabolic regulators [73,74,75]. This response aims to restore ETC integrity, stabilize protein folding, and prevent propagation of mitochondrial stress to the broader cellular environment.

In DS, however, UPRmt engagement appears early but functionally incomplete. In the Ts2Cje model, perinatal activation of an ATF5/GRP75-dependent program in the frontal cortex is accompanied by reduced expression of other stress mediators, including ATF4, CHOP, and SIRT3 [30,76,77]. This partial activation suggests that mitochondria in DS detect proteotoxic stress yet fail to mount a fully coordinated protective response. Such an imbalance may limit stabilization of respiratory chain complexes and compromise mitochondrial proteome maintenance under sustained oxidative conditions [1]. Consequently, incomplete UPRmt engagement may contribute to persistent mitochondrial fragility during neurodevelopmental windows when bioenergetic precision is essential.

When UPRmt buffering proves insufficient, cells rely increasingly on broader MQC mechanisms—including biogenesis, fusion–fission dynamics, and mitophagy—to maintain a functional mitochondrial pool [76]. In DS, evidence indicates that these compensatory systems are chronically stressed and frequently maladaptive, favoring accumulation rather than efficient turnover of dysfunctional organelles.

Impaired OXPHOS is a consistent and defining feature of DS biology. Across human tissues, patient-derived cells, and mouse models, trisomy 21 is associated with ETC inefficiency, particularly involving complex I deficits, reduced bioenergetic reserve, and increased ROS generation [60,64,72,77,78,79,80,81]. Some evidence suggests that trisomic cells downregulate OXPHOS as an adaptive attempt to limit electron leak and oxidative damage [82]. While this strategy may transiently reduce ROS production, it compromises ATP availability and diminishes metabolic flexibility, particularly under fluctuating energy demands.

In the developing and adult brain—where synaptic plasticity and circuit refinement require rapid, localized ATP production—chronic OXPHOS dampening may have profound consequences for neuronal resilience. In the Ts2Cje frontal cortex, dysfunction of complexes I and IV emerges early during developmental stages characterized by maximal synaptogenesis [77]. Moreover, lifespan-dependent remodeling of OXPHOS subunits—where some components are upregulated and others downregulated—suggests instability in ETC assembly and turnover under persistent proteostatic pressure [56,77]. Proteomic profiling of the human DS frontal cortex with and without AD neuropathology consistently identifies mitochondrial and energy-related pathways among the most altered signatures [44], underscoring the centrality of bioenergetic stress across DS and DSAD trajectories. Emerging evidence also implicates complex IV as a metabolic bottleneck, with upstream metabolic inhibitors converging on cytochrome c oxidase function [83].

ETC complexes are multi-subunit structures requiring coordinated nuclear and mitochondrial gene expression, import, folding, and assembly. Under chronic proteotoxic and oxidative stress, this coordination becomes destabilized. Thus, OXPHOS inefficiency in DS is both a consequence and an amplifier of mitochondrial proteostasis failure, reinforcing a feed-forward loop of ROS production, energetic insufficiency, and progressive proteostatic collapse in the brain.

### 3.2. Mitochondrial Quality Control (MQC) in Down Syndrome

MQC integrates mitochondrial biogenesis, fusion–fission dynamics, and mitophagy to preserve organelle integrity under stress [84]. In DS, converging data indicate that MQC is persistently challenged, particularly in neural tissue where spatially restricted ATP production and tight redox regulation are critical [59,60]. Morphologically, fragmentation of the mitochondrial network and cristae disorganization reflect imbalance in fusion–fission regulation [59,81,85]. Reduced fusion competence (MFN2/OPA1 dysfunction and altered OPA1 processing) combined with enhanced DRP1-mediated fission promotes respiratory inefficiency and ROS amplification [59,77,80,86,87]. Notably, experimental normalization of mitochondrial dynamics improves bioenergetics and supports neurogenic programs in DS models, confirming that dynamics are functionally causal rather than epiphenomenal [78,86,87,88,89,90]. Mitophagy impairment further exacerbates mitochondrial accumulation. Evidence of defective PINK1/Parkin signaling, incomplete autophagic flux, and inefficient removal of damaged mitochondria indicate compromised turnover capacity [77,91,92]. Persistent mTOR hyperactivation—reported early and sustained in the DS brain—restrains ULK1-mediated initiation of autophagy and mitophagy [77,91,92,93], thereby linking nutrient-sensing dysregulation to defective mitochondrial clearance. ULK1-dependent phosphorylation of mitophagy receptors such as FUNDC1 [93] provides a mechanistic bridge between mTOR status and cargo recruitment, reinforcing the notion that mTOR dysregulation [63,94,95,96,97] directly impairs mitochondrial quality control. Simultaneously, dysregulation of mitonuclear signaling pathways—including AMPK–PGC-1α–NRF1–TFAM—limits mitochondrial renewal and mtDNA maintenance under sustained stress [59,77,90]. Trisomy-linked gene dosage further compounds MQC dysfunction. Overexpression of ETS2 promotes mitochondrial apoptotic signaling in DS neuronal models [98], while intersectin-1 (ITSN1) influences mitochondrial death pathways via trafficking modules [99]. Repression of PGC-1α activity through DYRK1A/RCAN1–calcineurin/NFAT signaling [100,101,102] and NRIP1 overexpression [103] constrains mitochondrial biogenesis. Additionally, HSA21-encoded microRNAs—such as miR-155-5p targeting TFAM [104] and let-7c-5p potentially targeting ANT1/SLC25A4 [105]—further destabilize mitochondrial maintenance. Collectively, these trisomy-driven inputs create an imbalance wherein mitochondrial damage accumulates faster than MQC systems can compensate, accelerating redox stress and proteostatic vulnerability.

### 3.3. Insulin Resistance as a Proteostasis–Redox Switch in the DS Brain

In the CNS, insulin signaling functions as a metabolic rheostat that integrates nutrient sensing with mitochondrial competence, redox control, and proteostasis [106,107,108,109]. Through the IRS–PI3K–AKT cascade, insulin promotes metabolic flexibility, restrains excessive ROS generation, and modulates mTOR-dependent translation and autophagy pathways [106,107,108,109]. In DS, this insulin–mTOR axis is disrupted early and prominently in the brain [44,63,64,72,92,94,102]. Aberrant activation of PI3K/AKT/mTOR signaling has been documented in post-mortem DS tissue [91,92,94], reinforcing mTOR hyperactivation as a core trisomy-linked stress signature. Sustained mTOR activity suppresses ULK1-dependent autophagy initiation, impairs mitophagy, and favors persistence of ROS-generating mitochondria [91,92,94]. Brain insulin resistance (BIR) likely contributes upstream to this maladaptive state [63,64,72], linking metabolic signaling failure to mitochondrial dysfunction and oxidative damage. Neuronal-derived extracellular vesicles from young individuals with DS reveal coordinated insulin pathway disruption and aberrant mTOR activity [63], suggesting that this imbalance is developmentally established. O-GlcNAcylation adds an additional nutrient-sensitive regulatory layer. Reduced glucose uptake and insulin resistance decrease hexosamine biosynthetic pathway flux and UDP-GlcNAc availability, lowering protein O-GlcNAcylation and altering the balance between phosphorylation and adaptive stress signaling. In DS models, O-GlcNAc dysregulation coexists with OXPHOS defects and oxidative stress [110]. Taken together, these findings position insulin resistance not merely as a metabolic comorbidity but as a proteostasis–redox switch that shapes mitochondrial resilience and neurodegenerative vulnerability across the DS lifespan.

## 4. Ubiquitin–Proteasome System and Autophagy Dysfunction in Down Syndrome Brain

The ubiquitin–proteasome system (UPS) is the principal intracellular machinery responsible for selective protein degradation and dynamic regulation of protein turnover [111,112]. Oxidative modification of proteins can facilitate proteasomal recognition through partial unfolding, functionally linking redox balance to proteolytic efficiency [113]. A landmark study by our group provided the first comprehensive characterization of the polyubiquitinylation profile in the DS frontal cortex before and after Alzheimer neuropathology. Isolation of polyubiquitinated proteins from postmortem samples revealed a profound age-dependent remodeling of the ubiquitome [114]. Proteins displaying aberrant ubiquitin tagging were predominantly involved in protein quality control and energy metabolism, and oxidative modifications were closely associated with altered ubiquitin conjugation patterns [114,115]. Functional analyses further revealed significant reductions in proteasomal catalytic activities—including chymotrypsin-like, trypsin-like, and caspase-like activities in the DS frontal cortex, supporting impaired substrate clearance during early disease stages [114]. Similar reductions in proteasomal activity and increased ubiquitin-positive aggregates have been reported in DS fibroblasts and in the cerebellum of Ts65Dn mice [23,116]. Mechanistically, oxidative post-translational damage represents a major contributor to UPS dysfunction in DS. Redox proteomics analyses identified ubiquitin carboxyl-terminal hydrolase L1 (UCH-L1), a neuron-enriched deubiquitinating enzyme essential for ubiquitin recycling, as an early target of oxidative modification in the DS brain [23]. Subsequent studies demonstrated aberrant polyubiquitinylation of UCH-L1, suggesting structural instability and functional impairment [117]. Given its role in maintaining free ubiquitin pools, oxidative inactivation of UCH-L1 may further compromise proteasomal efficiency. Similar carbonylation-associated loss of UCH-L1 activity has been described in AD brain, reinforcing the pathogenic relevance of this mechanism [118].

Increased oxidation of SOD1, a feature observed in the DS brain, has been linked to inhibition of proteasome activity, further supporting direct interference of chronic redox imbalance with proteolytic capacity [115,118]. In addition to oxidative mechanisms, gene dosage effects inherent to trisomy 21 may contribute to UPS perturbation. Chromosome 21 encodes multiple UPS regulators, including deubiquitinating enzymes such as USP25 and USP16, as well as proteins involved in ubiquitin conjugation and proteasome assembly [119]. Although the direct functional consequences of their triplication remain incompletely defined, altered expression of UPS-related genes may destabilize ubiquitin homeostasis in the DS brain. Furthermore, triplication of BACH1 perturbs the nuclear BACH1/NRF2 balance, impairing antioxidant responses and amplifying oxidative stress [41,42,120]. Given the sensitivity of proteasome function to redox status, BACH1 gene dosage represents an important indirect contributor to UPS vulnerability. Altogether, enzymatic impairment, oxidative damage, and gene dosage imbalance converge to compromise ubiquitin-dependent proteolysis in DS. As proteasomal degradation becomes progressively insufficient, cells increasingly rely on complementary degradative pathways, particularly the autophagy lysosomal system, to maintain proteostasis.

Autophagy is a highly conserved degradation pathway responsible for the clearance of long-lived proteins, aggregates, and damaged organelles, including mitochondria and ER [121]. Through autophagosome formation and lysosomal fusion, autophagy ensures cytoplasmic quality control and metabolic adaptation under stress [122]. Its activity is tightly regulated by nutrient-sensitive signaling pathways, most prominently the mTOR axis [123]. In DS, accumulating evidence indicates profound and early autophagy dysregulation. Post-mortem DS frontal cortex exhibits hyperactivation of the PI3K/AKT/mTOR pathway associated with reduced autophagosome formation and altered expression of autophagy-related proteins [92]. Notably, mTORC1 hyperactivation is detectable during prenatal and early postnatal development in the DS hippocampus and persists across the lifespan [91], indicating chronic suppression of autophagic induction. These alterations correlate with increased Aβ accumulation and tau phosphorylation [124,125]. Experimental models reproduce these findings. Ts1Cje and Ts65Dn mice show reduced autophagic flux, altered LC3 processing, and dysregulation of Atg proteins, together with accumulation of pathogenic protein species [94,126,127]. Similarly, DS-derived fibroblasts display defective macroautophagy and impaired PINK1/PARKIN-dependent mitophagy [92]. Pharmacological inhibition of mTOR restores autophagic flux and partially rescues mitochondrial defects [96,128,129]. Additional trisomy-linked mechanisms further constrain autophagic efficiency. APP triplication and accumulation of its C99 fragment impair lysosomal acidification through interference with the V0-ATPase proton pump [130]. Oxidative modifications of autophagy-related and lysosomal proteins—including V0-ATPase and cathepsin D—have been documented in the DS brain and are associated with impaired LC3-II formation [23]. Chronic inflammatory signaling also contributes to autophagy modulation [6]. Collectively, autophagy impairment in DS is early, persistent, and multifactorial, contributing to accumulation of misfolded proteins and dysfunctional organelles.

**Figure 2:**
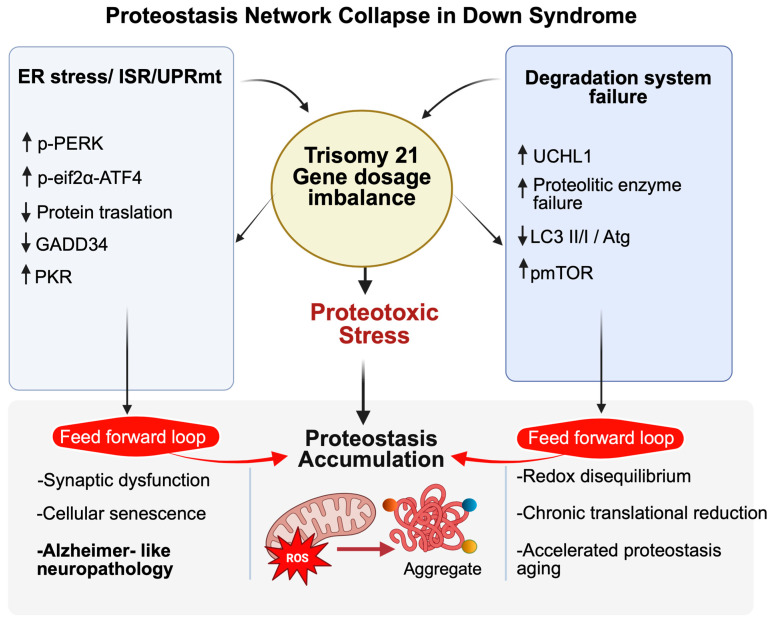
Schematic representation of proteostasis network collapse in Down syndrome. Trisomy 21-driven gene dosage imbalance perturbs multiple proteostasis modules, including endoplasmic reticulum (ER) stress and integrated stress responses (ISR/UPRmt), with increased PERK–eIF2α–ATF4 signaling, reduced global protein translation. Concomitantly, degradation pathways are impaired, as indicated by altered UCHL1 levels, defective proteolytic enzyme activity, and dysregulated autophagy (LC3 II/I and Atg proteins), leading to inefficient clearance of damaged proteins. Together, these alterations converge in proteotoxic stress and progressive accumulation of misfolded/aggregated proteins, which in turn sustain “feed forward” loops that promote synaptic dysfunction, cellular senescence, redox disequilibrium, accelerated proteostasis aging, and Alzheimer like neuropathology in Down syndrome.

Maintenance of proteostasis depends on coordinated activity of UPS and autophagy within an integrated degradation network. In the DS brain, dysfunction of these systems appears synergistic rather than compensatory. Reduced proteasomal activity and oxidative modifications in proteasome components [115] coexist with chronic mTOR hyperactivation and lysosomal impairment suppressing autophagic flux [92,94]. Proteomic analyses further reveal widespread alterations in proteins involved in degradation and redox regulation [44]. Ubiquitin signaling constitutes a central convergence point. When proteasomal degradation is insufficient, ubiquitinated substrates are redirected toward selective autophagy via adaptor proteins such as p62/SQSTM1 [131]. However, in DS—where autophagy is simultaneously compromised—this compensatory mechanism becomes ineffective, leading to accumulation of ubiquitinated aggregates [132]. Redox imbalance further amplifies this collapse. Oxidative stress directly damages proteasome subunits and autophagy machinery [133], while defective mitophagy sustains mitochondrial ROS production [44]. Thus, oxidative stress acts both upstream and downstream of proteostasis dysfunction, reinforcing a feed-forward cycle of degradation failure. Within this integrated framework, coordinated impairment of UPS and autophagy emerges as a central driver of proteostasis exhaustion and accelerated Alzheimer-like neurodegeneration in Down syndrome, providing a strong mechanistic rationale for therapeutic strategies aimed at restoring global degradative capacity (Figure 2).

## 5. Therapeutic Implications

The central involvement of degradation systems in DS proteostasis imbalance identifies multiple therapeutic entry points. Rather than targeting individual pathogenic proteins, increasing evidence supports strategies aimed at restoring global proteostasis capacity and redox resilience through modulation of interconnected stress-response and clearance pathways. Several studies demonstrated the ability of different compounds to target components of the proteostasis network in both preclinical and clinical studies.

### 5.1. Autophagy Modulation

Autophagy has emerged as a pivotal node linking mitochondrial dysfunction, proteotoxic stress, and neurodegeneration in DS. Several pharmacological interventions targeting the Akt/mTOR axis have demonstrated the ability to restore impaired autophagic flux and improve protein clearance [92,134,135]. In DS models, inhibition of mTOR signaling consistently reactivates autophagy and ameliorates downstream neuropathology. Aminooxyacetic acid (AOAA), a cystathionine β-synthase inhibitor, corrects CBS/H_2_S pathway overactivity in Dp(17)3Yey/+ mice, improving cognition and synaptic ATP production while attenuating ER stress, gliosis, and autophagic alterations [136]. Rapamycin represents the most extensively studied autophagy modulator in DS. Intranasal rapamycin administration in Ts65Dn mice normalizes hippocampal and cortical mTOR signaling, restores autophagic flux (increased LC3-II and autophagy-related proteins), improves insulin signaling, reduces APP processing and tau hyperphosphorylation, and rescues hippocampal-dependent memory [96,129]. Prenatal rapamycin treatment in Ts1Cje mice corrects synaptic plasticity abnormalities and spine morphology alterations, highlighting the developmental sensitivity of mTOR-dependent mechanisms [134]. Similarly, short-term intraperitoneal rapamycin restores spatial long-term memory in Ts1Cje mice, indirectly implicating autophagy induction in cognitive rescue [137]. Second-generation mTOR inhibitors such as AZD8055 restore macroautophagy and PINK1/PARKIN-dependent mitophagy in DS fibroblasts, rescuing mitochondrial clearance [92,134]. Metformin likewise improves mitophagy and lysosomal function in trisomic cells [81,92], reinforcing the therapeutic tractability of nutrient-sensing pathways. Additional interventions converge on mitochondrial-autophagy crosstalk. Polydatin reactivates mitochondrial bioenergetics and mitophagy while preventing premature senescence via modulation of microRNA-155 in T21 fibroblasts [88]. Intranasal Thiamet G restores O-GlcNAcylation, enhances autophagy induction, and rescues mitochondrial function in DS models [110]. The KYCCSRK peptide, derived from Biliverdin Reductase-A, restores brain insulin signaling, reduces oxidative damage, and limits amyloidogenic processing, indirectly reinforcing proteostasis capacity [138]. Collectively, these findings indicate that carefully titrated activation of autophagy and mitophagy represents a promising strategy to counteract degradation insufficiency in DS.

### 5.2. ISR and UPR Modulation

Chronic activation of the integrated stress response (ISR), characterized by persistent eIF2α phosphorylation, contributes to translational repression and impaired synaptic plasticity in DS [31]. Pharmacological inhibition of PKR using small-molecule inhibitors (PKRi) or early postnatal fluoxetine administration restores hippocampal neurogenesis and long-term memory in Ts65Dn mice [139,140,141]. ISRIB, a potent eIF2B activator, rescues de novo protein synthesis and improves memory performance in DS models [31], directly demonstrating the pathological relevance of sustained ISR activation. Targeting the PERK arm of the UPR has also shown therapeutic promise. Intranasal administration of the PERK inhibitor GSK2606414 in Ts2Cje mice reduces chronic PERK signaling, restores translational balance, and reactivates Nrf2-dependent antioxidant responses by correcting the Nrf2/Bach1 imbalance [30]. Beyond ER stress, early mitochondrial stress responses (UPRmt) have emerged as additional targets. Altered ATF5/GRP75 signaling in the Ts2Cje frontal cortex contributes to early oxidative distress [77], identifying mitochondrial proteostasis pathways as potential intervention nodes.

### 5.3. Ubiquitin–Proteasome System (UPS) Targeting

Given early proteasomal impairment in the DS brain, modulation of the UPS represents an additional therapeutic frontier. Trisomy 21 includes genes encoding deubiquitinases such as USP16 and USP25. Inhibition of USP16 rescues stem cell proliferation defects in DS models [142,143,144], while USP25 overexpression has been implicated in impaired neurogenesis and cognitive deficits, nominating it as a potential pharmaceutical target [145]. Importantly, crosstalk between UPS and autophagy suggests that enhancing autophagic clearance may relieve proteasomal burden. Intranasal rapamycin reduces accumulation of Lys63-linked polyubiquitinated proteins in Ts65Dn mice without affecting Lys48 linkages, indicating selective rebalancing of degradation pathways [97]. These findings support a systems-level approach in which coordinated restoration of UPS and autophagy may prove more effective than isolated pathway targeting.

### 5.4. Antioxidant and Redox-Modulating Strategies

Given the central role of oxidative stress in destabilizing proteostasis, antioxidant-based approaches have long been explored in DS. Preclinical studies demonstrated beneficial effects of α-tocopherol supplementation in Ts65Dn mice, including reduced lipid peroxidation, attenuation of cholinergic degeneration, and improved spatial memory [146,147,148]. However, large randomized clinical trials in adults with DS and AD failed to show significant cognitive benefit despite multi-year antioxidant administration [149]. Coenzyme Q10 improved oxidative DNA damage markers in children with DS, although long-term efficacy remained inconsistent [150,151]. Among polyphenols, epigallocatechin gallate (EGCG) has received the most attention. In Ts65Dn mice, EGCG restores neurogenesis, rebalances excitatory/inhibitory transmission, and improves learning [146,152,153,154,155,156]. Clinical trials in young adults demonstrated modest improvements in memory and executive function after 12 months of treatment [141,153,154,155,156]. Beyond DYRK1A inhibition, EGCG also modulates epigenetic regulators such as DNMT1 and ADAR1 and influences homocysteine metabolism and MMP/TIMP balance, potentially affecting amyloid dynamics [157]. Other flavonoids, including apigenin and 7,8-dihydroxyflavone (7,8-DHF), improve neurodevelopmental outcomes and mitochondrial bioenergetics in DS models [158,159,160,161]. Melatonin reduces oxidative damage and improves spatial learning in Ts65Dn mice [162,163,164].

Metformin modulates AMPK/NF-κB signaling and mitigates oxidative stress [165], while lithium restores REST levels and reduces oxidative damage in DS-derived neurons [166]. CAPE and its analogue VP961 restore the BACH1/NRF2 axis, promoting ARE-driven cytoprotective gene expression [41]. GLP-1 receptor agonists have also shown neuroprotective and antioxidant effects. The GLP-1 cleavage product improves hippocampal LTP and cognition in Ts65Dn mice [167], and other GLP-1RAs activate the Nrf2/HO-1 axis in neurodegenerative models [168], providing a rationale for further exploration in DS (Table 1 and Table 2).

**Figure 3:**
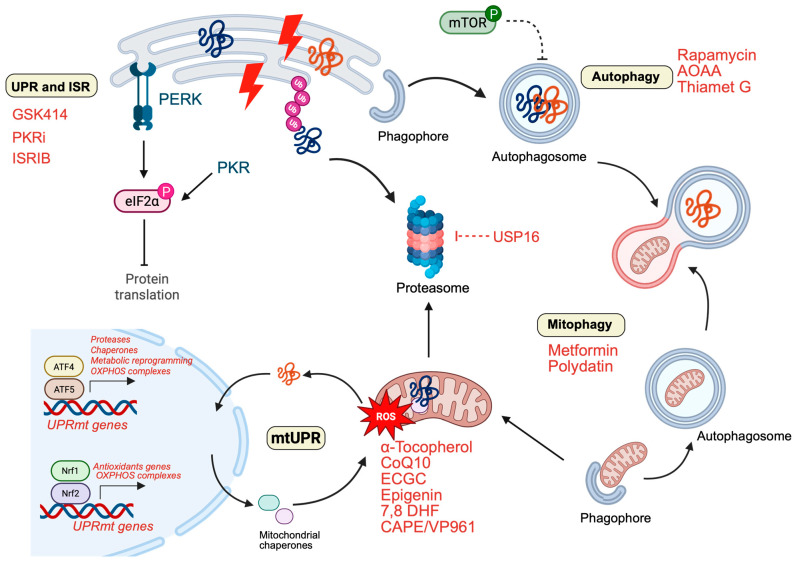
Major components of the proteostasis network altered in Down syndrome and their pharmacological modulators. In the autophagy panel (right), inhibition of mTOR by rapamycin, AOAA, and Thiamet G restores autophagic flux, promoting autophagosome formation and maturation into autolysosomes. Metformin and polydatin enhance mitophagy, facilitating the selective removal of damaged mitochondria. In the ER-stress and ISR/UPR panel (upper left), activation of PERK and PKR leads to eIF2α phosphorylation and suppression of global protein translation; GSK2606414, PKRi, and ISRIB are shown as pharmacological modulators that attenuate stress signaling and help rebalance protein synthesis. The central proteasome panel highlights the role of the deubiquitinase USP16 in proteasomal dysfunction and the accumulation of ubiquitinated substrates, emphasizing the ubiquitin–proteasome system as a critical hub of proteostasis control. In the mitochondrial UPR and redox panel (lower left), mitochondrial stress and excessive reactive oxygen species (ROS) production are counteracted by α-tocopherol, CoQ10, EGCG, apigenin, 7,8-dihydroxyflavone, and CAPE/VP961, which support mitochondrial proteostasis and antioxidant responses. Together, these pathways illustrate a network-based therapeutic strategy aimed at restoring autophagy and mitophagy, ISR/UPR signaling, ubiquitin–proteasome function, and redox homeostasis to improve cellular proteostasis in Down syndrome.

Taken together, therapeutic evidence in DS increasingly supports a shift from single-target interventions toward strategies aimed at restoring integrated proteostasis networks. Modulation of mTOR signaling, ISR/UPR pathways, mitochondrial quality control, UPS components, and redox balance converges on a shared objective: re-establishing cellular buffering capacity against chronic proteotoxic and oxidative stress (Figure 3). Because proteostasis failure in DS is developmentally established and progressively amplified across the lifespan, timing of intervention may be critical. Early modulation of stress-response and degradative pathways could potentially delay or attenuate the trajectory toward Alzheimer-like neurodegeneration.

## 6. Concluding Remarks

DS should no longer be conceptualized solely as a gene-dosage disorder, but rather as a systems-level perturbation of proteostasis networks established from early development. Trisomy 21 imposes chronic translational pressure, redox disequilibrium, and degradation pathway insufficiency that progressively erode cellular resilience across the lifespan. Sustained ER stress with PERK-dominant signaling, incomplete mitochondrial stress adaptation, impaired ubiquitin–proteasome activity, and suppressed autophagic flux converge into a coordinated failure of proteostasis. Rather than isolated defects, these alterations form an interconnected vulnerability network in which mitochondrial dysfunction, oxidative stress, and degradation insufficiency reinforce one another in a feed-forward manner. Within this framework, insulin resistance and chronic mTOR hyperactivation function as metabolic switches that lock trisomic cells into maladaptive stress signaling states, limiting adaptive clearance responses and amplifying proteotoxic burden. Alzheimer-like neuropathology in DS thus emerges not simply as a consequence of APP triplication, but as the late manifestation of decades-long proteostasis exhaustion. This integrated perspective reframes DS as a human model of premature aging, providing unique insight into the mechanistic interface between redox imbalance, mitochondrial fragility, and neurodegeneration. Therapeutic strategies aimed at restoring global proteostasis capacity rather than targeting individual aggregates may offer broader and more durable benefits.

Future research should prioritize longitudinal systems-level approaches to identify biomarkers of proteostasis resilience and define optimal timing for intervention. In doing so, DS may serve not only as a target for precision therapeutic development, but also as a translational framework for understanding the broader biology of age-related neurodegeneration.

## Figures and Tables

**Table 1:** List of all the compounds used in DS neuropathology pre-clinical studies.

Compound	Target	Study Type	Dosage	Length of the Treatment	Administration Route	Model	Ref.	Outcomes
**Unfolded Protein Response and Integrated Stress Response inhibitors**								
GSK2606414	PERK	Preclinical study	0.1 μg/μL	5 days (1× day)	intranasal treatment	Ts2Cje	[30]	Restored protein synthesis; reduced OS
ISRIB	eiF2a	Preclinical study	2.5 mg/kg	7 days (once every 2 days)	i.p. injection	Ts65dn	[31]	Restored protein synthesis; improved long-term memory
PKRi	PKR	Preclinical study	0.1 mg/kg	6 days (1× day)	i.p. injection	Ts65dn	[139,140,141]	Rescued long-term memory and synaptic plasticity
Fluoxetine	PKR (indirect)	Preclinical study	Not specified	Early postnatal	Systemic	Ts65dn	[139,140,141]	Rescued long-term memory; neurogenesis
**UPS modulators**								
USP16	USP16	Preclinical study	Not specified	Not specified	In vitro	Ts65Dn DS stem cells	[142,143,144]	Rescued proliferation defects
Rapamycin	mTOR	Preclinical study	1 μg	90 days (1× day, 3× week)	Intranasal	Ts65Dn	[96,129]	Reduced Lys63-linked polyubiquitinated proteins
**Autophagy modulators**								
AOAA	CBS/H_2_S pathway	Preclinical study	1 mg/kg/day	14 days, daily administration	intraperitoneally	Dp(17)3Yey/+	[136]	Improved cognition; restored autophagy
Rapamycin	mTOR	Preclinical study	1 μg	90 days (1× day, 3× week)	Intranasal	Ts65Dn	[96,129]	Reduced APP/tau pathology; rescued hippocampal tasks
		Preclinical study	1 mg/kg	3 consecutive days during gestation	i.p. injection to pregnant dams	Ts1Cje	[134]	Corrected synaptic plasticity
		Preclinical study	10 mg/kg	5 days (1× day)	i.p. injection	Ts1Cje	[137]	Restored spatial long-term memory
AZD8055	mTORC1/2	Preclinical study	0.1 μM	2, 4 and 8 h	In vitro	Human fibroblasts	[92]	Restored autophagy and mitophagy
Metformin	AMPK/mTOR	Preclinical study	0.5 mM	72 h	In vitro	Human T21 fibroblasts	[81,92]	Restored mitophagy and lysosomal clearance
Polydatin	Mitophagy; miR-155	Preclinical study	10 μM	24–72 h	In vitro	Human T21 fibroblasts	[88]	Mitochondrial bioenergetics and mitophagy
KYCCSRK peptide	BVR-A	Preclinical study	0.5 mM	2 weeks	Intranasal	Ts2Cje	[138]	Restored insulin signaling and mitochondrial function
Thiamet G	O-GlcNAcylation	Preclinical study	25 μg	5 days (2 × day)	Intranasal	Ts2Cje	[110]	Boosted autophagy induction
**Antioxidants**								
α-Tocopherol	ROS	Preclinical study	50 mg/Kg	5 months	diet supplementation	Ts65dn	[146]	Reduced OS; improved spatial working memory
		Preclinical study	0.1% *w*/*w* for Kg of diet	Pregnancy and pups	maternal supplementation	Ts65dn	[148]	Improved cognition; reduced lipid peroxidation
Apigenin	NF-κB; antioxidant	Preclinical study	2 μM (in vitro); 200–250 mg/kg/day (in vivo)	Prenatal + postnatal	Oral/systemic	T21 amniocytes; Ts1Cje	[158,159]	Reduced OS; improved spatial memory (sex-specific)
7,8-DHF	TrkB (BDNF mimetic)	Preclinical study	5 mg/kg	Postnatal treatment: for 12 days, Adult treatment for 40 days.	Subcutaneous administration	Ts65Dn	[158]	Restored mitochondrial bioenergetics; increased
Melatonin	ROS scavenger	Preclinical study	10 mg/kg/die	5 months	Oral	Ts65Dn	[162,163,164]	Improved spatial learning; reduced lipid peroxidation
			10 mg/kg/die	6 months	Oral	Ts65Dn	[162,163,164]	Reduced OS and hippocampal senescence
Metformin	AMPK/NF-κB	Preclinical study	10, 30, 50 μM	48 h	Systemic	Human T21 fibroblasts	[165,166]	Mitigated oxidative damage
Lithium	REST	Preclinical study	10 mM	24 h	In vitro	iPSC-derived neurons	[166]	Restored REST levels; reduced OS
CAPE	BACH1/NRF2	Preclinical study	10 μM	6 h	In vitro	human DS lymphoblastoid (LCLs)	[41]	Promoted NRF2 activation
VP961	BACH1/NRF2	Preclinical study	5 μM	6 h	In vitro	human DS lymphoblastoid (LCLs)	[41]	Promoted NRF2 activation
GLP-1 (cleavage product)	GLP-1R; mitochondrial ROS	Preclinical study	500 ng/g	2–3 weeks	Ip injection	9 mo Ts65Dn	[167]	Decreased mitochondrial OS
EGCG	DYRK1A; ROS	Preclinical study	20 μM	72 h (changed every 24 h)	cells treatment	Human DS cell cultures	[152]	Reduced OS and mitochondrial energy deficit
		Preclinical study	2–3 mg/day	1 month	water supplementation	Ts65Dn/TgDyrk1A	[154]	Improved cognition
		Preclinical study	225 mg/kg/day	4 weeks	water supplementation	Ts65Dn	[156]	Restored excitatory/inhibitory (E/I) imbalance (GABA modulation)
		Preclinical study	25 mg/Kg/day	P3 to P15	subcutaneous injection	Ts65Dn	[169]	Restored neurogenesis at P15; no cognitive improvement at P45
		Preclinical study	30 mg/kg/day	30 days	water supplementation	Ts65Dn	[153]	Rescued CA1 dendritic spine density, improved cognition
		Preclinical study	50 mg/kg	T1 (21 days) T2 (mating until 90 days) T3(P60–P90)	diet supplementation	Dp(16)1Yey	[155]	Rescued GAD67; restored VGAT1/VGLUT1 balance; improved novel object recognition memory

**Table 2:** List of all the compounds used in DS neuropathology in clinical studies.

Compound	Target	Study Type	Dosage	Length of the Treatment	Administration Route	Model	Ref.	Outcomes
**Antioxidants**								
		Clinical study	900 IU + ascorbic acid (200 mg) + α-lipoic acid (600 mg)	2 years (daily)	oral	DS and AD individuals	[147]	No cognition improvement
		Clinical study	1000 IU	over 3 years (twice daily)	oral	DS over 50 years	[170]	No cognitive improvement
		Clinical study	266 mg + α-lipoic acid (100 mg/day)	4 months (daily)	oral	DS children	[171]	Reduced OS at DNA level
		Clinical study	400 mg + Vitamin C (500 mg/day)	over 6 months (daily)	oral	DS children and teenagers	[172]	Reduced blood levels of lipid peroxidation
CoQ10	Mitochondrial ETC	Clinical study	4 mg/kg/day	6 or 20 months (daily)	oral	Children DS	[151]	Inhibited DNA oxidative damage; inconsistent long-term effects
		Clinical study	4 mg/kg/day	4-year (daily)	oral	Children DS	[150]	No reduced OS level level at RNA or DNA level
EGCG		Clinical study	9 mg/kg/day	6 and 12 months	diet supplementation	Young adults with DS	[154,173]	Reduced plasma homocysteine; rescued cognitive performances

## Data Availability

No new data were created or analyzed in this study.

## Abbreviations

The following abbreviations are used in this manuscript:

**AD**	Alzheimer’s disease
**AMPK**	AMP-activated protein kinase
**ANT1**	Adenine nucleotide translocator 1
**AOAA**	Aminooxyacetic acid
**APP**	Amyloid precursor protein
**ARE**	Antioxidant response element
**ATF4**	Activating transcription factor 4
**ATF5**	Activating transcription factor 5
**ATF6**	Activating transcription factor 6 (sensore UPR)
**ATP**	Adenosine triphosphate
**Aβ (A-beta)**	Amyloid-β/Amiloide-beta
**BACH1**	BTB and CNC homology 1
**BCL-2**	B-cell lymphoma 2
**BIR**	Brain insulin resistance
**C99**	C-terminal 99-amino acid APP fragment
**CAPE**	Caffeic acid phenethyl ester
**CBS**	Cystathionine β-synthase
**CHOP**	C/EBP homologous protein
**Chr21**	Chromosome 21
**CNS**	Central nervous system
**CoQ10**	Coenzyme Q10
**DNMT1**	DNA methyltransferase 1
**DRP1**	Dynamin-related protein 1
**DS**	Down syndrome
**DSAD**	Down syndrome with Alzheimer’s disease neuropathology
**DYRK1A**	Dual-specificity tyrosine-regulated kinase 1A
**EGCG**	Epigallocatechin gallate
**eIF2α (eIF2-alpha)**	Eukaryotic initiation factor 2 alpha
**eIF2B**	Eukaryotic initiation factor 2B
**EOAD**	Early-onset Alzheimer’s disease
**ER**	Endoplasmic reticulum
**ETC**	Electron transport chain
**ETS2**	ETS proto-oncogene 2
**FUNDC1**	FUN14 domain containing 1
**GADD34**	Growth arrest and DNA damage-inducible protein 34
**GLP-1**	Glucagon-like peptide-1
**GLP-1RA**	GLP-1 receptor agonist
**GRP75**	75 kDa glucose-regulated protein
**GRP78/BiP**	78 kDa glucose-regulated protein/Binding immunoglobulin protein
**H_2_S**	Hydrogen sulfide
**HO-1**	Heme oxygenase 1
**Hsa21**	Homo sapiens chromosome 21
**HSP70**	Heat shock protein 70
**iPSC**	Induced pluripotent stem cell
**IRE1**	Inositol-requiring enzyme 1
**IRS**	Insulin receptor substrate
**ISR**	Integrated stress response
**LC3**	Microtubule-associated protein 1 light chain 3
**LTP**	Long-term potentiation
**MFN2**	Mitofusin 2
**miR-155-5p**	microRNA-155-5p
**MMP**	Matrix metalloproteinase
**MQC**	Mitochondrial quality control
**mtDNA**	Mitochondrial DNA
**mTOR**	mammalian target of rapamycin
**mTORC1**	mTOR complex 1
**NF-κB (NF-kappa-B)**	Nuclear factor kappa-light-chain-enhancer of activated B cells
**NRF1**	Nuclear respiratory factor 1
**NRIP1**	Nuclear receptor interacting protein 1
**Nrf2**	Nuclear factor erythroid 2-related factor 2
**OPA1**	Optic atrophy 1
**OXPHOS**	Oxidative phosphorylation
**p16^INK4a^**	Cyclin-dependent kinase inhibitor 2A
**p21**	Cyclin-dependent kinase inhibitor 1
**p53**	Tumor protein p53
**p62/SQSTM1**	Sequestosome 1
**PBMC**	Peripheral blood mononuclear cell
**PERK**	Protein kinase R-like ER kinase
**PET**	Positron emission tomography
**PGC-1α (PGC-1-alpha)**	Peroxisome proliferator-activated receptor gamma coactivator 1-alpha
**PI3K**	Phosphoinositide 3-kinase
**PINK1**	PTEN-induced kinase 1
**PKR**	Protein kinase R (EIF2AK2)
**PKRi**	PKR inhibitor
**RCAN1**	Regulator of calcineurin 1
**REST**	RE1-silencing transcription factor
**RNS**	Reactive nitrogen species
**ROS**	Reactive oxygen species
**SASP**	Senescence-associated secretory phenotype
**SIRT3**	Sirtuin 3
**SLC25A4**	Solute carrier family 25 member 4 (ANT1)
**SOD1**	Superoxide dismutase 1 (Cu/Zn-SOD)
**TFAM**	Mitochondrial transcription factor A
**TIMP**	Tissue inhibitor of metalloproteinases
**UCH-L1**	Ubiquitin carboxyl-terminal hydrolase L1
**UDP-GlcNAc**	Uridine diphosphate N-acetylglucosamine
**ULK1**	Unc-51-like kinase 1
**UPR**	Unfolded protein response
**UPR^mt^**	Mitochondrial unfolded protein response
**UPS**	Ubiquitin–proteasome system
**USP16**	Ubiquitin-specific peptidase 16
**USP25**	Ubiquitin-specific peptidase 25
**V0-ATPase**	Vacuolar-type H^+^-ATPase

## References

[1] Antonarakis S.E., Skotko B.G., Rafii M.S., Strydom A., Pape S.E., Bianchi D.W., Sherman S.L., Reeves R.H. (2020). Down syndrome. Nat. Rev. Dis. Primers.

[2] Chou C.Y., Liu L.Y., Chen C.Y., Tsai C.H., Hwa H.L., Chang L.Y., Lin Y.S., Hsieh F.J. (2008). Gene expression variation increase in trisomy 21 tissues. Mamm. Genome.

[3] Zhegalova I.V., Vasiluev P.A., Flyamer I.M., Shtompel A.S., Glazyrina E., Shilova N., Minzhenkova M., Markova Z., Petrova N.V., Dashinimaev E.B. (2023). Trisomies Reorganize Human 3D Genome. Int. J. Mol. Sci..

[4] Reichard J., Zimmer-Bensch G. (2021). The Epigenome in Neurodevelopmental Disorders. Front. Neurosci..

[5] Ippolito M.R., Zerbib J., Eliezer Y., Reuveni E., Vigano S., De Feudis G., Shulman E.D., Savir Kadmon A., Slutsky R., Chang T. (2024). Increased RNA and Protein Degradation Is Required for Counteracting Transcriptional Burden and Proteotoxic Stress in Human Aneuploid Cells. Cancer Discov..

[6] Waugh K.A., Minter R., Baxter J., Chi C., Galbraith M.D., Tuttle K.D., Eduthan N.P., Kinning K.T., Andrysik Z., Araya P. (2023). Triplication of the interferon receptor locus contributes to hallmarks of Down syndrome in a mouse model. Nat. Genet..

[7] Tcw J., Goate A.M. (2017). Genetics of beta-Amyloid Precursor Protein in Alzheimer’s Disease. Cold Spring Harb. Perspect. Med..

[8] Wiseman F.K., Pulford L.J., Barkus C., Liao F., Portelius E., Webb R., Chavez-Gutierrez L., Cleverley K., Noy S., Sheppard O. (2018). Trisomy of human chromosome 21 enhances amyloid-beta deposition independently of an extra copy of APP. Brain.

[9] Fortea J., Zaman S.H., Hartley S., Rafii M.S., Head E., Carmona-Iragui M. (2021). Alzheimer’s disease associated with Down syndrome: A genetic form of dementia. Lancet Neurol..

[10] Gomez W., Morales R., Maracaja-Coutinho V., Parra V., Nassif M. (2020). Down syndrome and Alzheimer’s disease: Common molecular traits beyond the amyloid precursor protein. Aging.

[11] Zong Y., Li H., Liao P., Chen L., Pan Y., Zheng Y., Zhang C., Liu D., Zheng M., Gao J. (2024). Mitochondrial dysfunction: Mechanisms and advances in therapy. Signal Transduct. Target. Ther..

[12] Shreeya T., Ansari M.S., Kumar P., Saifi M., Shati A.A., Alfaifi M.Y., Elbehairi S.E.I. (2023). Senescence: A DNA damage response and its role in aging and Neurodegenerative Diseases. Front. Aging.

[13] Labbadia J., Morimoto R.I. (2015). The biology of proteostasis in aging and disease. Annu. Rev. Biochem..

[14] Zhu J., Wu C., Yang L. (2024). Cellular senescence in Alzheimer’s disease: From physiology to pathology. Transl. Neurodegener..

[15] Sparks L.D., Kryscio R.J., Hunsaker J.C. (2013). Early age-related progression of AD-like neuropathology in Down’s syndrome. Am. J. Neurodegener. Dis..

[16] Qu H.Q., Liu Y., Connolly J.J., Mentch F.D., Kao C., Hakonarson H. (2025). Risk of Alzheimer’s disease in Down syndrome: Insights gained by multi-omics. Alzheimers Dement..

[17] Boyle R., Koops E.A., Ances B., Andrews E.J., Arenaza-Urquijo E.M., Bejanin A., Brickman A.M., Buckley R.F., Clas G.S., Costello E. (2025). Resistance and resilience to Alzheimer’s disease in Down syndrome. Alzheimers Dement..

[18] Kocaturk N.M., Gozuacik D. (2018). Crosstalk Between Mammalian Autophagy and the Ubiquitin-Proteasome System. Front. Cell Dev. Biol..

[19] Pytel D., Fromm Longo J. (2025). The Proteostasis Network in Proteinopathies: Mechanisms and Interconnections. Am. J. Pathol..

[20] Coleman R.A., Johnson M.E., Konopka A., Chew Y.L. (2026). Proteostasis, disease and the ageing neuron: Compartmental complexity in non-renewing cells. Ageing Res. Rev..

[21] Zhu J., Tsai H.J., Gordon M.R., Li R. (2018). Cellular Stress Associated with Aneuploidy. Dev. Cell.

[22] Aivazidis S., Coughlan C.M., Rauniyar A.K., Jiang H., Liggett L.A., Maclean K.N., Roede J.R. (2017). The burden of trisomy 21 disrupts the proteostasis network in Down syndrome. PLoS ONE.

[23] Di Domenico F., Coccia R., Cocciolo A., Murphy M.P., Cenini G., Head E., Butterfield D.A., Giorgi A., Schinina M.E., Mancuso C. (2013). Impairment of proteostasis network in Down syndrome prior to the development of Alzheimer’s disease neuropathology: Redox proteomics analysis of human brain. Biochim. Biophys. Acta.

[24] Hohn A., Tramutola A., Cascella R. (2020). Proteostasis Failure in Neurodegenerative Diseases: Focus on Oxidative Stress. Oxid. Med. Cell Longev..

[25] Di Domenico F., Lanzillotta C. (2022). The disturbance of protein synthesis/degradation homeostasis is a common trait of age-related neurodegenerative disorders. Adv. Protein Chem. Struct. Biol..

[26] Nagar P., Sharma P., Dhapola R., Kumari S., Medhi B., HariKrishnaReddy D. (2023). Endoplasmic reticulum stress in Alzheimer’s disease: Molecular mechanisms and therapeutic prospects. Life Sci..

[27] Sidhom E., O’Brien J.T., Butcher A.J., Smith H.L., Mallucci G.R., Underwood B.R. (2022). Targeting the Unfolded Protein Response as a Disease-Modifying Pathway in Dementia. Int. J. Mol. Sci..

[28] Bravo R., Parra V., Gatica D., Rodriguez A.E., Torrealba N., Paredes F., Wang Z.V., Zorzano A., Hill J.A., Jaimovich E. (2013). Endoplasmic reticulum and the unfolded protein response: Dynamics and metabolic integration. Int. Rev. Cell Mol. Biol..

[29] Lanzillotta C., Di Domenico F. (2021). Stress Responses in Down Syndrome Neurodegeneration: State of the Art and Therapeutic Molecules. Biomolecules.

[30] Lanzillotta C., Zuliani I., Tramutola A., Barone E., Blarzino C., Folgiero V., Caforio M., Valentini D., Villani A., Locatelli F. (2021). Chronic PERK induction promotes Alzheimer-like neuropathology in Down syndrome: Insights for therapeutic intervention. Prog. Neurobiol..

[31] Zhu P.J., Khatiwada S., Cui Y., Reineke L.C., Dooling S.W., Kim J.J., Li W., Walter P., Costa-Mattioli M. (2019). Activation of the ISR mediates the behavioral and neurophysiological abnormalities in Down syndrome. Science.

[32] Perluigi M., Di Domenico F., Butterfield D.A. (2024). Oxidative damage in neurodegeneration: Roles in the pathogenesis and progression of Alzheimer disease. Physiol. Rev..

[33] Hetz C., Saxena S. (2017). ER stress and the unfolded protein response in neurodegeneration. Nat. Rev. Neurol..

[34] Costa-Mattioli M., Walter P. (2020). The integrated stress response: From mechanism to disease. Science.

[35] Ma Y., Hendershot L.M. (2003). Delineation of a negative feedback regulatory loop that controls protein translation during endoplasmic reticulum stress. J. Biol. Chem..

[36] Fawcett T.W., Martindale J.L., Guyton K.Z., Hai T., Holbrook N.J. (1999). Complexes containing activating transcription factor (ATF)/cAMP-responsive-element-binding protein (CREB) interact with the CCAAT/enhancer-binding protein (C/EBP)-ATF composite site to regulate Gadd153 expression during the stress response. Biochem. J..

[37] Hoozemans J.J., van Haastert E.S., Nijholt D.A., Rozemuller A.J., Eikelenboom P., Scheper W. (2009). The unfolded protein response is activated in pretangle neurons in Alzheimer’s disease hippocampus. Am. J. Pathol..

[38] Hoozemans J.J., Veerhuis R., Van Haastert E.S., Rozemuller J.M., Baas F., Eikelenboom P., Scheper W. (2005). The unfolded protein response is activated in Alzheimer’s disease. Acta Neuropathol..

[39] Scheper W., Nijholt D.A., Hoozemans J.J. (2011). The unfolded protein response and proteostasis in Alzheimer disease: Preferential activation of autophagy by endoplasmic reticulum stress. Autophagy.

[40] Ma T., Trinh M.A., Wexler A.J., Bourbon C., Gatti E., Pierre P., Cavener D.R., Klann E. (2013). Suppression of eIF2alpha kinases alleviates Alzheimer’s disease-related plasticity and memory deficits. Nat. Neurosci..

[41] Pagnotta S., Tramutola A., Barone E., Di Domenico F., Pittala V., Salerno L., Folgiero V., Caforio M., Locatelli F., Petrini S. (2022). CAPE and its synthetic derivative VP961 restore BACH1/NRF2 axis in Down Syndrome. Free Radic. Biol. Med..

[42] Di Domenico F., Pupo G., Mancuso C., Barone E., Paolini F., Arena A., Blarzino C., Schmitt F.A., Head E., Butterfield D.A. (2015). Bach1 overexpression in Down syndrome correlates with the alteration of the HO-1/BVR-a system: Insights for transition to Alzheimer’s disease. J. Alzheimers Dis..

[43] Buttari B., Tramutola A., Rojo A.I., Chondrogianni N., Saha S., Berry A., Giona L., Miranda J.P., Profumo E., Davinelli S. (2025). Proteostasis Decline and Redox Imbalance in Age-Related Diseases: The Therapeutic Potential of NRF2. Biomolecules.

[44] Di Domenico F., Greco V., Tramutola A., Rataj-Baniowska M., Barone E., Lanzillotta C., Pieroni L., Butterfield D.A., Herault Y., Pagnotta S. (2025). Proteome Signature of Alzheimer-Like Phenotypes in Frontal Cortices From Young and Old Individuals With Down Syndrome. Mol. Neurobiol..

[45] Rastogi M., Bartolucci M., Nanni M., Aloisio M., Vozzi D., Petretto A., Contestabile A., Cancedda L. (2024). Integrative multi-omic analysis reveals conserved cell-projection deficits in human Down syndrome brains. Neuron.

[46] Farrell C., Buhidma Y., Mumford P., Heywood W.E., Hallqvist J., Flores-Aguilar L., Andrews E.J., Rahimzadah N., Taso O.S., Doran E. (2025). Apolipoprotein E abundance is elevated in the brains of individuals with Down syndrome-Alzheimer’s disease. Acta Neuropathol..

[47] Marta-Ariza M., Leitner D.F., Kanshin E., Suazo J., Giusti Pedrosa A., Thierry M., Lee E.B., Devinsky O., Drummond E., Fortea J. (2025). Comparison of the amyloid plaque proteome in Down syndrome, early-onset Alzheimer’s disease, and late-onset Alzheimer’s disease. Acta Neuropathol..

[48] Lanzillotta C., Greco V., Valentini D., Villani A., Folgiero V., Caforio M., Locatelli F., Pagnotta S., Barone E., Urbani A. (2020). Proteomics Study of Peripheral Blood Mononuclear Cells in Down Syndrome Children. Antioxidants.

[49] Botte A., Laine J., Xicota L., Heiligenstein X., Fontaine G., Kasri A., Rivals I., Goh P., Faklaris O., Cossec J.C. (2020). Ultrastructural and dynamic studies of the endosomal compartment in Down syndrome. Acta Neuropathol. Commun..

[50] Liu Y., Borel C., Li L., Muller T., Williams E.G., Germain P.L., Buljan M., Sajic T., Boersema P.J., Shao W. (2017). Systematic proteome and proteostasis profiling in human Trisomy 21 fibroblast cells. Nat. Commun..

[51] Granholm A.C. (2025). Vertebrate and Invertebrate Animal Models for the Study of Down Syndrome. Int. J. Mol. Sci..

[52] Lanzillotta C., Tramutola A., Meier S., Schmitt F., Barone E., Perluigi M., Di Domenico F., Abisambra J.F. (2018). Early and Selective Activation and Subsequent Alterations to the Unfolded Protein Response in Down Syndrome Mouse Models. J. Alzheimers Dis..

[53] Peng L., Baradar A.A., Aguado J., Wolvetang E. (2023). Cellular senescence and premature aging in Down Syndrome. Mech. Ageing Dev..

[54] Roth G.M., Sun B., Greensite F.S., Lott I.T., Dietrich R.B. (1996). Premature aging in persons with Down syndrome: MR findings. AJNR Am. J. Neuroradiol..

[55] Duchon A., Del Mar Muniz Moreno M., Chevalier C., Nalesso V., Andre P., Fructuoso-Castellar M., Mondino M., Po C., Noblet V., Birling M.C. (2022). Ts66Yah, a mouse model of Down syndrome with improved construct and face validity. Dis. Model. Mech..

[56] Lanzillotta C., Baniowska M.R., Prestia F., Sette C., Nalesso V., Perluigi M., Barone E., Duchon A., Tramutola A., Herault Y. (2024). Shaping down syndrome brain cognitive and molecular changes due to aging using adult animals from the Ts66Yah murine model. Neurobiol. Dis..

[57] Ludwig M.P., Galbraith M.D., Eduthan N.P., Hill A.A., Clay M.R., Tellez C.M., Wilky B.A., Elias A., Espinosa J.M., Sullivan K.D. (2023). Proteasome Inhibition Sensitizes Liposarcoma to MDM2 Inhibition with Nutlin-3 by Activating the ATF4/CHOP Stress Response Pathway. Cancer Res..

[58] Song J., Herrmann J.M., Becker T. (2021). Quality control of the mitochondrial proteome. Nat. Rev. Mol. Cell Biol..

[59] Zuo X. (2025). Mitochondrial Imbalance in Down Syndrome: A Driver of Accelerated Brain Aging?. Aging Dis..

[60] Valenti D., Vacca R.A. (2023). Brain Mitochondrial Bioenergetics in Genetic Neurodevelopmental Disorders: Focus on Down, Rett and Fragile X Syndromes. Int. J. Mol. Sci..

[61] Palozza P., Barone E., Mancuso C., Picci N. (2008). The protective role of carotenoids against 7-keto-cholesterol formation in solution. Mol. Cell Biochem..

[62] Barone E., Head E., Butterfield D.A., Perluigi M. (2017). HNE-modified proteins in Down syndrome: Involvement in development of Alzheimer disease neuropathology. Free Radic. Biol. Med..

[63] Perluigi M., Picca A., Montanari E., Calvani R., Marini F., Matassa R., Tramutola A., Villani A., Familiari G., Domenico F.D. (2022). Aberrant crosstalk between insulin signaling and mTOR in young Down syndrome individuals revealed by neuronal-derived extracellular vesicles. Alzheimers Dement..

[64] Lanzillotta C., Tramutola A., Di Giacomo G., Marini F., Butterfield D.A., Di Domenico F., Perluigi M., Barone E. (2021). Insulin resistance, oxidative stress and mitochondrial defects in Ts65dn mice brain: A harmful synergistic path in down syndrome. Free Radic. Biol. Med..

[65] Mollo N., Cicatiello R., Aurilia M., Scognamiglio R., Genesio R., Charalambous M., Paladino S., Conti A., Nitsch L., Izzo A. (2020). Targeting Mitochondrial Network Architecture in Down Syndrome and Aging. Int. J. Mol. Sci..

[66] Valenti D., de Bari L., De Filippis B., Henrion-Caude A., Vacca R.A. (2014). Mitochondrial dysfunction as a central actor in intellectual disability-related diseases: An overview of Down syndrome, autism, Fragile X and Rett syndrome. Neurosci. Biobehav. Rev..

[67] Valenti D., Braidy N., De Rasmo D., Signorile A., Rossi L., Atanasov A.G., Volpicella M., Henrion-Caude A., Nabavi S.M., Vacca R.A. (2018). Mitochondria as pharmacological targets in Down syndrome. Free Radic. Biol. Med..

[68] Ganguly B.B., Kadam N.N. (2023). Therapeutics for mitochondrial dysfunction-linked diseases in Down syndrome. Mitochondrion.

[69] Zamponi E., Helguera P.R. (2019). The Shape of Mitochondrial Dysfunction in Down Syndrome. Dev. Neurobiol..

[70] Stagni F., Giacomini A., Emili M., Guidi S., Bartesaghi R. (2018). Neurogenesis impairment: An early developmental defect in Down syndrome. Free Radic. Biol. Med..

[71] Uguagliati B., Stagni F., Emili M., Giacomini A., Russo C., Guidi S., Bartesaghi R. (2022). Early Appearance of Dendritic Alterations in Neocortical Pyramidal Neurons of the Ts65Dn Model of Down Syndrome. Dev. Neurosci..

[72] Tramutola A., Lanzillotta C., Di Domenico F., Head E., Butterfield D.A., Perluigi M., Barone E. (2020). Brain insulin resistance triggers early onset Alzheimer disease in Down syndrome. Neurobiol. Dis..

[73] Shpilka T., Haynes C.M. (2018). The mitochondrial UPR: Mechanisms, physiological functions and implications in ageing. Nat. Rev. Mol. Cell Biol..

[74] Martinus R.D., Garth G.P., Webster T.L., Cartwright P., Naylor D.J., Hoj P.B., Hoogenraad N.J. (1996). Selective induction of mitochondrial chaperones in response to loss of the mitochondrial genome. Eur. J. Biochem..

[75] Zhao Q., Wang J., Levichkin I.V., Stasinopoulos S., Ryan M.T., Hoogenraad N.J. (2002). A mitochondrial specific stress response in mammalian cells. EMBO J..

[76] Eckl E.M., Ziegemann O., Krumwiede L., Fessler E., Jae L.T. (2021). Sensing, signaling and surviving mitochondrial stress. Cell Mol. Life Sci..

[77] Lanzillotta S., Esteve D., Lanzillotta C., Tramutola A., Lloret A., Forte E., Pesce V., Picca A., Di Domenico F., Perluigi M. (2025). Altered mitochondrial unfolded protein response and protein quality control promote oxidative distress in down syndrome brain. Free Radic. Biol. Med..

[78] Scala I., Valenti D., Scotto D’Aniello V., Marino M., Riccio M.P., Bravaccio C., Vacca R.A., Strisciuglio P. (2021). Epigallocatechin-3-Gallate Plus Omega-3 Restores the Mitochondrial Complex I and F(0)F(1)-ATP Synthase Activities in PBMCs of Young Children with Down Syndrome: A Pilot Study of Safety and Efficacy. Antioxidants.

[79] Vacca R.A., Valenti D. (2015). Green tea EGCG plus fish oil omega-3 dietary supplements rescue mitochondrial dysfunctions and are safe in a Down’s syndrome child. Clin. Nutr..

[80] Valenti D., Rossi L., Marzulli D., Bellomo F., De Rasmo D., Signorile A., Vacca R.A. (2017). Inhibition of Drp1-mediated mitochondrial fission improves mitochondrial dynamics and bioenergetics stimulating neurogenesis in hippocampal progenitor cells from a Down syndrome mouse model. Biochim. Biophys. Acta Mol. Basis Dis..

[81] Izzo A., Nitti M., Mollo N., Paladino S., Procaccini C., Faicchia D., Cali G., Genesio R., Bonfiglio F., Cicatiello R. (2017). Metformin restores the mitochondrial network and reverses mitochondrial dysfunction in Down syndrome cells. Hum. Mol. Genet..

[82] Helguera P., Seiglie J., Rodriguez J., Hanna M., Helguera G., Busciglio J. (2013). Adaptive downregulation of mitochondrial function in down syndrome. Cell Metab..

[83] Petrosino M., Zuhra K., Kieronska-Rudek A., Janickova L., Bremer O., Khalaf M., Logue B.A., Szabo C. (2025). Cyanide overproduction impairs cellular bioenergetics in Down syndrome. Neurotherapeutics.

[84] Ye L., Fu X., Li Q. (2025). Mitochondrial Quality Control in Health and Disease. MedComm.

[85] Lanzillotta S., Rolfi L.R., Zulli B., Barone E. (2026). Metabolic breakdown: Linking insulin resistance and mitochondrial dysfunction to neurodegeneration in Alzheimer’s disease. Neural Regen. Res..

[86] Mollo N., Nitti M., Zerillo L., Faicchia D., Micillo T., Accarino R., Secondo A., Petrozziello T., Cali G., Cicatiello R. (2019). Pioglitazone Improves Mitochondrial Organization and Bioenergetics in Down Syndrome Cells. Front. Genet..

[87] Piccoli C., Izzo A., Scrima R., Bonfiglio F., Manco R., Negri R., Quarato G., Cela O., Ripoli M., Prisco M. (2013). Chronic pro-oxidative state and mitochondrial dysfunctions are more pronounced in fibroblasts from Down syndrome foeti with congenital heart defects. Hum. Mol. Genet..

[88] Valenti D., Abbrescia D.I., Marzano F., Ravagnan G., Tullo A., Vacca R.A. (2025). Polydatin reactivates mitochondrial bioenergetics and mitophagy while preventing premature senescence by modulating microRNA-155 and its direct targets in human fibroblasts with trisomy 21. Free Radic. Biol. Med..

[89] Valenti D., Stagni F., Emili M., Guidi S., Bartesaghi R., Vacca R.A. (2021). Impaired Brain Mitochondrial Bioenergetics in the Ts65Dn Mouse Model of Down Syndrome Is Restored by Neonatal Treatment with the Polyphenol 7,8-Dihydroxyflavone. Antioxidants.

[90] Lanzillotta C., Prestia F., Greco V., Iavarone F., Cordella F., Sette C., Forte E., Tramutola A., Lanzillotta S., Cassano T. (2025). Enhancing protein O-GlcNAcylation in down syndrome mice mitigates memory dysfunctions through the rescue of mitochondrial bioenergetics, stress responses and pathological markers. Redox Biol..

[91] Iyer A.M., van Scheppingen J., Milenkovic I., Anink J.J., Adle-Biassette H., Kovacs G.G., Aronica E. (2014). mTOR Hyperactivation in down syndrome hippocampus appears early during development. J. Neuropathol. Exp. Neurol..

[92] Bordi M., Darji S., Sato Y., Mellen M., Berg M.J., Kumar A., Jiang Y., Nixon R.A. (2019). mTOR hyperactivation in Down Syndrome underlies deficits in autophagy induction, autophagosome formation, and mitophagy. Cell Death Dis..

[93] Wu W., Tian W., Hu Z., Chen G., Huang L., Li W., Zhang X., Xue P., Zhou C., Liu L. (2014). ULK1 translocates to mitochondria and phosphorylates FUNDC1 to regulate mitophagy. EMBO Rep..

[94] Perluigi M., Pupo G., Tramutola A., Cini C., Coccia R., Barone E., Head E., Butterfield D.A., Di Domenico F. (2014). Neuropathological role of PI3K/Akt/mTOR axis in Down syndrome brain. Biochim. Biophys. Acta.

[95] Perluigi M., Di Domenico F., Barone E., Butterfield D.A. (2021). mTOR in Alzheimer disease and its earlier stages: Links to oxidative damage in the progression of this dementing disorder. Free Radic. Biol. Med..

[96] Di Domenico F., Tramutola A., Barone E., Lanzillotta C., Defever O., Arena A., Zuliani I., Foppoli C., Iavarone F., Vincenzoni F. (2019). Restoration of aberrant mTOR signaling by intranasal rapamycin reduces oxidative damage: Focus on HNE-modified proteins in a mouse model of down syndrome. Redox Biol..

[97] Tramutola A., Lanzillotta C., Arena A., Barone E., Perluigi M., Di Domenico F. (2016). Increased Mammalian Target of Rapamycin Signaling Contributes to the Accumulation of Protein Oxidative Damage in a Mouse Model of Down’s Syndrome. Neurodegener. Dis..

[98] Helguera P., Pelsman A., Pigino G., Wolvetang E., Head E., Busciglio J. (2005). ets-2 promotes the activation of a mitochondrial death pathway in Down’s syndrome neurons. J. Neurosci..

[99] Predescu S.A., Predescu D.N., Knezevic I., Klein I.K., Malik A.B. (2007). Intersectin-1s regulates the mitochondrial apoptotic pathway in endothelial cells. J. Biol. Chem..

[100] Arron J.R., Winslow M.M., Polleri A., Chang C.P., Wu H., Gao X., Neilson J.R., Chen L., Heit J.J., Kim S.K. (2006). NFAT dysregulation by increased dosage of DSCR1 and DYRK1A on chromosome 21. Nature.

[101] Handschin C., Rhee J., Lin J., Tarr P.T., Spiegelman B.M. (2003). An autoregulatory loop controls peroxisome proliferator-activated receptor gamma coactivator 1alpha expression in muscle. Proc. Natl. Acad. Sci. USA.

[102] Dierssen M., Fructuoso M., Martinez de Lagran M., Perluigi M., Barone E. (2020). Down Syndrome Is a Metabolic Disease: Altered Insulin Signaling Mediates Peripheral and Brain Dysfunctions. Front. Neurosci..

[103] Izzo A., Manco R., Bonfiglio F., Cali G., De Cristofaro T., Patergnani S., Cicatiello R., Scrima R., Zannini M., Pinton P. (2014). NRIP1/RIP140 siRNA-mediated attenuation counteracts mitochondrial dysfunction in Down syndrome. Hum. Mol. Genet..

[104] Quinones-Lombrana A., Blanco J.G. (2015). Chromosome 21-derived hsa-miR-155-5p regulates mitochondrial biogenesis by targeting Mitochondrial Transcription Factor A (TFAM). Biochim. Biophys. Acta.

[105] Izzo A., Manco R., de Cristofaro T., Bonfiglio F., Cicatiello R., Mollo N., De Martino M., Genesio R., Zannini M., Conti A. (2017). Overexpression of Chromosome 21 miRNAs May Affect Mitochondrial Function in the Hearts of Down Syndrome Fetuses. Int. J. Genom..

[106] Neth B.J., Craft S. (2017). Insulin Resistance and Alzheimer’s Disease: Bioenergetic Linkages. Front. Aging Neurosci..

[107] Arnold S.E., Arvanitakis Z., Macauley-Rambach S.L., Koenig A.M., Wang H.Y., Ahima R.S., Craft S., Gandy S., Buettner C., Stoeckel L.E. (2018). Brain insulin resistance in type 2 diabetes and Alzheimer disease: Concepts and conundrums. Nat. Rev. Neurol..

[108] Tramutola A., Di Domenico F., Perluigi M., Barone E. (2025). Biliverdin reductase-A is a key modulator in insulin signaling and metabolism. Trends Endocrinol. Metab..

[109] Chen W., Cai W., Hoover B., Kahn C.R. (2022). Insulin action in the brain: Cell types, circuits, and diseases. Trends Neurosci..

[110] Zuliani I., Lanzillotta C., Tramutola A., Francioso A., Pagnotta S., Barone E., Perluigi M., Di Domenico F. (2021). The Dysregulation of OGT/OGA Cycle Mediates Tau and APP Neuropathology in Down Syndrome. Neurotherapeutics.

[111] Kwon Y.T., Ciechanover A. (2017). The Ubiquitin Code in the Ubiquitin-Proteasome System and Autophagy. Trends Biochem. Sci..

[112] Komander D., Rape M. (2012). The ubiquitin code. Annu. Rev. Biochem..

[113] Grimm S., Hohn A., Grune T. (2012). Oxidative protein damage and the proteasome. Amino Acids.

[114] Tramutola A., Perluigi M. (2021). Polyubiquitin Profile in Down Syndrome and Alzheimer’s Disease Brain. Methods Mol. Biol..

[115] Di Domenico F., Pupo G., Tramutola A., Giorgi A., Schinina M.E., Coccia R., Head E., Butterfield D.A., Perluigi M. (2014). Redox proteomics analysis of HNE-modified proteins in Down syndrome brain: Clues for understanding the development of Alzheimer disease. Free Radic. Biol. Med..

[116] Necchi D., Lomoio S., Scherini E. (2011). Dysfunction of the ubiquitin-proteasome system in the cerebellum of aging Ts65Dn mice. Exp. Neurol..

[117] Tramutola A., Di Domenico F., Barone E., Arena A., Giorgi A., di Francesco L., Schinina M.E., Coccia R., Head E., Butterfield D.A. (2017). Polyubiquitinylation Profile in Down Syndrome Brain Before and After the Development of Alzheimer Neuropathology. Antioxid. Redox Signal.

[118] Mi Z., Graham S.H. (2023). Role of UCHL1 in the pathogenesis of neurodegenerative diseases and brain injury. Ageing Res. Rev..

[119] Valero R., Marfany G., Gonzalez-Angulo O., Gonzalez-Gonzalez G., Puelles L., Gonzalez-Duarte R. (1999). USP25, a novel gene encoding a deubiquitinating enzyme, is located in the gene-poor region 21q11.2. Genomics.

[120] Dhakshinamoorthy S., Jain A.K., Bloom D.A., Jaiswal A.K. (2005). Bach1 competes with Nrf2 leading to negative regulation of the antioxidant response element (ARE)-mediated NAD(P)H:quinone oxidoreductase 1 gene expression and induction in response to antioxidants. J. Biol. Chem..

[121] Mizushima N., Levine B. (2020). Autophagy in Human Diseases. N. Engl. J. Med..

[122] Yim W.W., Mizushima N. (2020). Lysosome biology in autophagy. Cell Discov..

[123] Kim Y.C., Guan K.L. (2015). mTOR: A pharmacologic target for autophagy regulation. J. Clin. Investig..

[124] Colacurcio D.J., Pensalfini A., Jiang Y., Nixon R.A. (2018). Dysfunction of autophagy and endosomal-lysosomal pathways: Roles in pathogenesis of Down syndrome and Alzheimer’s Disease. Free Radic. Biol. Med..

[125] Tramutola A., Triplett J.C., Di Domenico F., Niedowicz D.M., Murphy M.P., Coccia R., Perluigi M., Butterfield D.A. (2015). Alteration of mTOR signaling occurs early in the progression of Alzheimer disease (AD): Analysis of brain from subjects with pre-clinical AD, amnestic mild cognitive impairment and late-stage AD. J. Neurochem..

[126] Troca-Marin J.A., Alves-Sampaio A., Montesinos M.L. (2011). An increase in basal BDNF provokes hyperactivation of the Akt-mammalian target of rapamycin pathway and deregulation of local dendritic translation in a mouse model of Down’s syndrome. J. Neurosci..

[127] Sheppard O., Plattner F., Rubin A., Slender A., Linehan J.M., Brandner S., Tybulewicz V.L., Fisher E.M., Wiseman F.K. (2012). Altered regulation of tau phosphorylation in a mouse model of down syndrome aging. Neurobiol. Aging.

[128] Urbano-Gamez J.D., Casanas J.J., Benito I., Montesinos M.L. (2021). Prenatal treatment with rapamycin restores enhanced hippocampal mGluR-LTD and mushroom spine size in a Down’s syndrome mouse model. Mol. Brain.

[129] Tramutola A., Lanzillotta C., Barone E., Arena A., Zuliani I., Mosca L., Blarzino C., Butterfield D.A., Perluigi M., Di Domenico F. (2018). Intranasal rapamycin ameliorates Alzheimer-like cognitive decline in a mouse model of Down syndrome. Transl. Neurodegener..

[130] Im E., Jiang Y., Stavrides P.H., Darji S., Erdjument-Bromage H., Neubert T.A., Choi J.Y., Wegiel J., Lee J.H., Nixon R.A. (2023). Lysosomal dysfunction in Down syndrome and Alzheimer mouse models is caused by v-ATPase inhibition by Tyr(682)-phosphorylated APP betaCTF. Sci. Adv..

[131] Kumar A.V., Mills J., Lapierre L.R. (2022). Selective Autophagy Receptor p62/SQSTM1, a Pivotal Player in Stress and Aging. Front. Cell Dev. Biol..

[132] Aivazidis S., Jain A., Rauniyar A.K., Anderson C.C., Marentette J.O., Orlicky D.J., Fritz K.S., Harris P.S., Siegel D., Maclean K.N. (2019). SNARE proteins rescue impaired autophagic flux in Down syndrome. PLoS ONE.

[133] Puente-Bedia A., Berciano M.T., Martinez-Cue C., Lafarga M., Rueda N. (2022). Oxidative-Stress-Associated Proteostasis Disturbances and Increased DNA Damage in the Hippocampal Granule Cells of the Ts65Dn Model of Down Syndrome. Antioxidants.

[134] Troca-Marin J.A., Casanas J.J., Benito I., Montesinos M.L. (2014). The Akt-mTOR pathway in Down’s syndrome: The potential use of rapamycin/rapalogs for treating cognitive deficits. CNS Neurol. Disord. Drug Targets.

[135] Alldred M.J., Chao H.M., Lee S.H., Beilin J., Powers B.E., Petkova E., Strupp B.J., Ginsberg S.D. (2019). Long-term effects of maternal choline supplementation on CA1 pyramidal neuron gene expression in the Ts65Dn mouse model of Down syndrome and Alzheimer’s disease. FASEB J..

[136] Panagaki T., Janickova L., Petrovic D., Zuhra K., Ditroi T., Juranyi E.P., Bremer O., Ascencao K., Philipp T.M., Nagy P. (2024). Neurobehavioral dysfunction in a mouse model of Down syndrome: Upregulation of cystathionine beta-synthase, H(2)S overproduction, altered protein persulfidation, synaptic dysfunction, endoplasmic reticulum stress, and autophagy. Geroscience.

[137] Andrade-Talavera Y., Benito I., Casanas J.J., Rodriguez-Moreno A., Montesinos M.L. (2015). Rapamycin restores BDNF-LTP and the persistence of long-term memory in a model of Down’s syndrome. Neurobiol. Dis..

[138] Tramutola A., Lanzillotta S., Aceto G., Pagnotta S., Ruffolo G., Cifelli P., Marini F., Ripoli C., Palma E., Grassi C. (2023). Intranasal Administration of KYCCSRK Peptide Rescues Brain Insulin Signaling Activation and Reduces Alzheimer’s Disease-like Neuropathology in a Mouse Model for Down Syndrome. Antioxidants.

[139] Bianchi P., Ciani E., Guidi S., Trazzi S., Felice D., Grossi G., Fernandez M., Giuliani A., Calza L., Bartesaghi R. (2010). Early pharmacotherapy restores neurogenesis and cognitive performance in the Ts65Dn mouse model for Down syndrome. J. Neurosci..

[140] Du R.H., Tan J., Sun X.Y., Lu M., Ding J.H., Hu G. (2016). Fluoxetine Inhibits NLRP3 Inflammasome Activation: Implication in Depression. Int. J. Neuropsychopharmacol..

[141] Jammi N.V., Whitby L.R., Beal P.A. (2003). Small molecule inhibitors of the RNA-dependent protein kinase. Biochem. Biophys. Res. Commun..

[142] Adorno M., Sikandar S., Mitra S.S., Kuo A., Nicolis Di Robilant B., Haro-Acosta V., Ouadah Y., Quarta M., Rodriguez J., Qian D. (2013). Usp16 contributes to somatic stem-cell defects in Down’s syndrome. Nature.

[143] Reinitz F., Chen E.Y., Nicolis di Robilant B., Chuluun B., Antony J., Jones R.C., Gubbi N., Lee K., Ho W.H.D., Kolluru S.S. (2022). Inhibiting USP16 rescues stem cell aging and memory in an Alzheimer’s model. eLife.

[144] Xu J.C., Dawson V.L., Dawson T.M. (2013). Usp16: Key controller of stem cells in Down syndrome. EMBO J..

[145] Cai F., Song B., Yang Y., Liao H., Li R., Wang Z., Cao R., Chen H., Wang J., Wu Y. (2023). USP25 contributes to defective neurogenesis and cognitive impairments. FASEB J..

[146] Lockrow J., Prakasam A., Huang P., Bimonte-Nelson H., Sambamurti K., Granholm A.C. (2009). Cholinergic degeneration and memory loss delayed by vitamin E in a Down syndrome mouse model. Exp. Neurol..

[147] Lott I.T., Doran E., Nguyen V.Q., Tournay A., Head E., Gillen D.L. (2011). Down syndrome and dementia: A randomized, controlled trial of antioxidant supplementation. Am. J. Med. Genet. A.

[148] Shichiri M., Yoshida Y., Ishida N., Hagihara Y., Iwahashi H., Tamai H., Niki E. (2011). alpha-Tocopherol suppresses lipid peroxidation and behavioral and cognitive impairments in the Ts65Dn mouse model of Down syndrome. Free Radic. Biol. Med..

[149] Mancuso C., Siciliano R., Barone E. (2011). Curcumin and Alzheimer disease: This marriage is not to be performed. J. Biol. Chem..

[150] Larsen E.L., Padella L., Bergholdt H.K.M., Henriksen T., Santoro L., Gabrielli O., Poulsen H.E., Littarru G.P., Orlando P., Tiano L. (2018). The effect of long-term treatment with coenzyme Q10 on nucleic acid modifications by oxidation in children with Down syndrome. Neurobiol. Aging.

[151] Tiano L., Padella L., Santoro L., Carnevali P., Principi F., Bruge F., Gabrielli O., Littarru G.P. (2012). Prolonged coenzyme Q10 treatment in Down syndrome patients: Effect on DNA oxidation. Neurobiol. Aging.

[152] Valenti D., De Rasmo D., Signorile A., Rossi L., de Bari L., Scala I., Granese B., Papa S., Vacca R.A. (2013). Epigallocatechin-3-gallate prevents oxidative phosphorylation deficit and promotes mitochondrial biogenesis in human cells from subjects with Down’s syndrome. Biochim. Biophys. Acta.

[153] Catuara-Solarz S., Espinosa-Carrasco J., Erb I., Langohr K., Notredame C., Gonzalez J.R., Dierssen M. (2015). Principal Component Analysis of the Effects of Environmental Enrichment and (-)-epigallocatechin-3-gallate on Age-Associated Learning Deficits in a Mouse Model of Down Syndrome. Front. Behav. Neurosci..

[154] De la Torre R., De Sola S., Pons M., Duchon A., de Lagran M.M., Farre M., Fito M., Benejam B., Langohr K., Rodriguez J. (2014). Epigallocatechin-3-gallate, a DYRK1A inhibitor, rescues cognitive deficits in Down syndrome mouse models and in humans. Mol. Nutr. Food Res..

[155] Souchet B., Duchon A., Gu Y., Dairou J., Chevalier C., Daubigney F., Nalesso V., Creau N., Yu Y., Janel N. (2019). Prenatal treatment with EGCG enriched green tea extract rescues GAD67 related developmental and cognitive defects in Down syndrome mouse models. Sci. Rep..

[156] Souchet B., Guedj F., Penke-Verdier Z., Daubigney F., Duchon A., Herault Y., Bizot J.C., Janel N., Creau N., Delatour B. (2015). Pharmacological correction of excitation/inhibition imbalance in Down syndrome mouse models. Front. Behav. Neurosci..

[157] Tyagi S.C., Smolenkova I., Zheng Y., Singh M. (2026). Epigenetic Control of Hyperuricemia and Gout by Gene Writer DNMT1 and RNA Editor ADAR1: Mechanism of Gout and Amyloid Dissolution in Down Syndrome. Biochem. Genet..

[158] Giacomini A., Stagni F., Emili M., Uguagliati B., Rimondini R., Bartesaghi R., Guidi S. (2019). Timing of Treatment with the Flavonoid 7,8-DHF Critically Impacts on Its Effects on Learning and Memory in the Ts65Dn Mouse. Antioxidants.

[159] Guedj F., Siegel A.E., Pennings J.L.A., Alsebaa F., Massingham L.J., Tantravahi U., Bianchi D.W. (2020). Apigenin as a Candidate Prenatal Treatment for Trisomy 21: Effects in Human Amniocytes and the Ts1Cje Mouse Model. Am. J. Hum. Genet..

[160] Amir M., Shafi S., Parveen S., Reshi A.A., Ahmad A. (2024). Network Pharmacology Identifies Intersection Genes of Apigenin and Naringenin in Down Syndrome as Potential Therapeutic Targets. Pharmaceuticals.

[161] Javadi B., Sobhani Z. (2024). Role of apigenin in targeting metabolic syndrome: A systematic review. Iran. J. Basic. Med. Sci..

[162] Corrales A., Martinez P., Garcia S., Vidal V., Garcia E., Florez J., Sanchez-Barcelo E.J., Martinez-Cue C., Rueda N. (2013). Long-term oral administration of melatonin improves spatial learning and memory and protects against cholinergic degeneration in middle-aged Ts65Dn mice, a model of Down syndrome. J. Pineal Res..

[163] Corrales A., Vidal R., Garcia S., Vidal V., Martinez P., Garcia E., Florez J., Sanchez-Barcelo E.J., Martinez-Cue C., Rueda N. (2014). Chronic melatonin treatment rescues electrophysiological and neuromorphological deficits in a mouse model of Down syndrome. J. Pineal Res..

[164] Parisotto E.B., Vidal V., Garcia-Cerro S., Lantigua S., Wilhelm Filho D., Sanchez-Barcelo E.J., Martinez-Cue C., Rueda N. (2016). Chronic Melatonin Administration Reduced Oxidative Damage and Cellular Senescence in the Hippocampus of a Mouse Model of Down Syndrome. Neurochem. Res..

[165] Buczynska A., Malinowski P., Zbikowski A., Kretowski A.J., Zbucka-Kretowska M. (2025). Metformin modulates oxidative stress via activation of AMPK/NF-kappaB signaling in Trisomy 21 fibroblasts: An in vitro study. Front. Mol. Biosci..

[166] Lam X.J., Maniam S., Ling K.H., Cheah P.S. (2025). Lithium restores nuclear REST and Mitigates oxidative stress in down syndrome iPSC-Derived neurons. Neuroscience.

[167] Day S.M., Yang W., Wang X., Stern J.E., Zhou X., Macauley S.L., Ma T. (2019). Glucagon-Like Peptide-1 Cleavage Product Improves Cognitive Function in a Mouse Model of Down Syndrome. eNeuro.

[168] Ghosh P., Fontanella R.A., Scisciola L., Pesapane A., Taktaz F., Franzese M., Puocci A., Ceriello A., Prattichizzo F., Rizzo M.R. (2023). Targeting redox imbalance in neurodegeneration: Characterizing the role of GLP-1 receptor agonists. Theranostics.

[169] Stagni F., Giacomini A., Emili M., Trazzi S., Guidi S., Sassi M., Ciani E., Rimondini R., Bartesaghi R. (2016). Short- and long-term effects of neonatal pharmacotherapy with epigallocatechin-3-gallate on hippocampal development in the Ts65Dn mouse model of Down syndrome. Neuroscience.

[170] Sano M., Aisen P.S., Andrews H.F., Tsai W.Y., Lai F., Dalton A.J., International Down S., Alzheimer’s Disease C. (2016). Vitamin E in aging persons with Down syndrome: A randomized, placebo-controlled clinical trial. Neurology.

[171] Mustafa Nachvak S., Reza Neyestani T., Ali Mahboob S., Sabour S., Ali Keshawarz S., Speakman J.R. (2014). alpha-Tocopherol supplementation reduces biomarkers of oxidative stress in children with Down syndrome: A randomized controlled trial. Eur. J. Clin. Nutr..

[172] Parisotto E.B., Garlet T.R., Cavalli V.L., Zamoner A., da Rosa J.S., Bastos J., Micke G.A., Frode T.S., Pedrosa R.C., Wilhelm Filho D. (2014). Antioxidant intervention attenuates oxidative stress in children and teenagers with Down syndrome. Res. Dev. Disabil..

[173] de la Torre R., de Sola S., Hernandez G., Farre M., Pujol J., Rodriguez J., Espadaler J.M., Langohr K., Cuenca-Royo A., Principe A. (2016). Safety and efficacy of cognitive training plus epigallocatechin-3-gallate in young adults with Down’s syndrome (TESDAD): A double-blind, randomised, placebo-controlled, phase 2 trial. Lancet Neurol..

